# Transcriptomic analysis of cave, surface, and hybrid samples of the isopod *Asellus aquaticus* and identification of chromosomal location of candidate genes for cave phenotype evolution

**DOI:** 10.1186/s13227-023-00213-z

**Published:** 2023-05-06

**Authors:** Haeli J. Lomheim, Lizet Reyes Rodas, Lubna Mulla, Layla Freeborn, Dennis A. Sun, Sheri A. Sanders, Meredith E. Protas

**Affiliations:** 1 Department of Natural Sciences and Mathematics, Dominican University of California, San Rafael, CA 94901 USA; 2 Center for Research Data and Digital Scholarship, University of Colorado Boulder, Boulder, CO 80309 USA; 3 Department of Molecular and Cell Biology, University of California at Berkeley, Berkeley, CA 94720 USA; 4 Department of Biological Sciences, University of Notre Dame, Notre Dame, IN 46556 USA; 5 Department of Biology, Georgetown University, Washington, DC 20057 USA

**Keywords:** Cave, Pigment, Eye, Troglomorphy, Allele-specific expression

## Abstract

**Background:**

Transcriptomic methods can be used to elucidate genes and pathways responsible for phenotypic differences between populations. *Asellus aquaticus* is a freshwater isopod crustacean with surface- and cave-dwelling ecomorphs that differ greatly in multiple phenotypes including pigmentation and eye size. Multiple genetic resources have been generated for this species, but the genes and pathways responsible for cave-specific characteristics have not yet been identified. Our goal was to generate transcriptomic resources in tandem with taking advantage of the species’ ability to interbreed and generate hybrid individuals.

**Results:**

We generated transcriptomes of the Rakov Škocjan surface population and the Rak Channel of Planina Cave population that combined Illumina short-read assemblies and PacBio Iso-seq long-read sequences. We investigated differential expression at two different embryonic time points as well as allele-specific expression of *F*_1_ hybrids between cave and surface individuals. RNAseq of *F*_2_ hybrids, as well as genotyping of a backcross, allowed for positional information of multiple candidate genes from the differential expression and allele-specific analyses.

**Conclusions:**

As expected, genes involved in phototransduction and ommochrome synthesis were under-expressed in the cave samples as compared to the surface samples. Allele-specific expression analysis of *F*_1_ hybrids identified genes with cave-biased (cave allele has higher mRNA levels than the surface allele) and surface-biased expression (surface allele has higher mRNA levels than the cave allele). RNAseq of *F*_2_ hybrids allowed for multiple genes to be placed to previously mapped genomic regions responsible for eye and pigmentation phenotypes. In the future, these transcriptomic resources will guide prioritization of candidates for functional analysis.

**Supplementary Information:**

The online version contains supplementary material available at 10.1186/s13227-023-00213-z.

## Background

Cave animals are fascinating organisms that can have striking features, such as eye and pigment loss. Over recent years, it has become possible to study cave animals whose genetics and genomes have never before been able to be studied (reviewed in [1-3]). This wealth of information has greatly increased our understanding of cave animals and cave-adapted characteristics.

A major challenge for this research is that information from genomic resources can be difficult to interpret and/or test if there is limited range of molecular methods that can be used in that particular species. As such, it is crucial to develop methods that allow for the interpretation of genomic information, including genetic maps or positional information from a genome, the ability to set up genetic crosses, comparative embryological methods, and tools for genetic perturbation.

Key to many of the above molecular methods is the presence of two forms (in our case, a cave and surface ecomorph) that can interbreed and produce fertile offspring. Unfortunately, this is a rare situation with the shining example being the cavefish, *Astyanax mexicanus*, which has served as an inspiration for what questions can be answered in a cave system. Genomes, transcriptomes, developmental methods, behavioral assays, CRISPR, RNAi and, numerous other methods and tools have been generated for this species, which allowed the amount of information and understanding of this species to grow tremendously (reviewed in [4]).

Other species with cave and surface ecomorphs that can interbreed in the laboratory could provide similar insight as *A. mexicanus* to the field of cave biology. More importantly, though, integrating the information from multiple species with cave-dwelling forms will provide a much more complete understanding of adaptation to the cave environment.

One of the species that has similar potential to *A. mexicanus* is *Asellus aquaticus*, an isopod crustacean found in Europe. *Asellus aquaticus* has been described as an eco-evolutionary model as well as an evo-devo model [5, 6]. There are multiple surface and cave populations of this system, many of which are thought to be independently colonized, which vary greatly in many phenotypes, including eye and pigment loss [7, 8]. Because it is possible to interbreed surface and cave populations in the lab, genetic crosses have been made and mapping studies have been performed identifying regions responsible for different eye and pigment phenotypes.

Though much information has been gained on this species, it is still unknown what the genes and mutations are behind the changes present in cave and surface populations. Toward this end, genomic information has been generated for this species including a draft genome and transcriptomes of multiple populations and different developmental time points [7, 9-11]. Though genomic and transcriptomic information has been a huge asset for this species, the sheer amount of information generated by these techniques is difficult to parse through to find pathways that are perturbed and causative genes.

Here, we address this challenge by generating transcriptomic data while also taking advantage of the ability of the animals to hybridize to narrow the list of candidate genes involved in traits, such as eye loss and pigment loss. We generated transcriptomes from the Slovenian Rak Channel of Planina Cave population and the Slovenian Rakov Škocjan surface population, hereby referred to as CAVE_rr and SURF_rs respectively, using Illumina and Iso-seq data. In addition, we examined differential expression of genes at two different embryonic timepoints in CAVE_rr and SURF_rs samples. Then, we took advantage of the ability of *A. aquaticus* to hybridize and expanded upon a previous study looking at allele-specific expression of the top 100 differentially expressed genes [9], examining allele-specific expression transcriptome-wide. In addition, we sequenced 15 *F*_2_ animals of different phenotypes and used them as a tool to gain positional information for genes present in the transcriptomes, relative to an existing linkage map. Finally, we placed additional candidates on the existing linkage map with the goal of seeing whether they coincided with known regions responsible for eye and pigment loss. We have thereby used the intersection of transcriptomic information and mapping techniques to narrow the list of candidate genes responsible for cave-specific phenotypes; these genes will be prioritized for future functional analysis.

## Results

### Cave and surface transcriptomes generated from Illumina and Iso-seq data

**Fig. 1:**
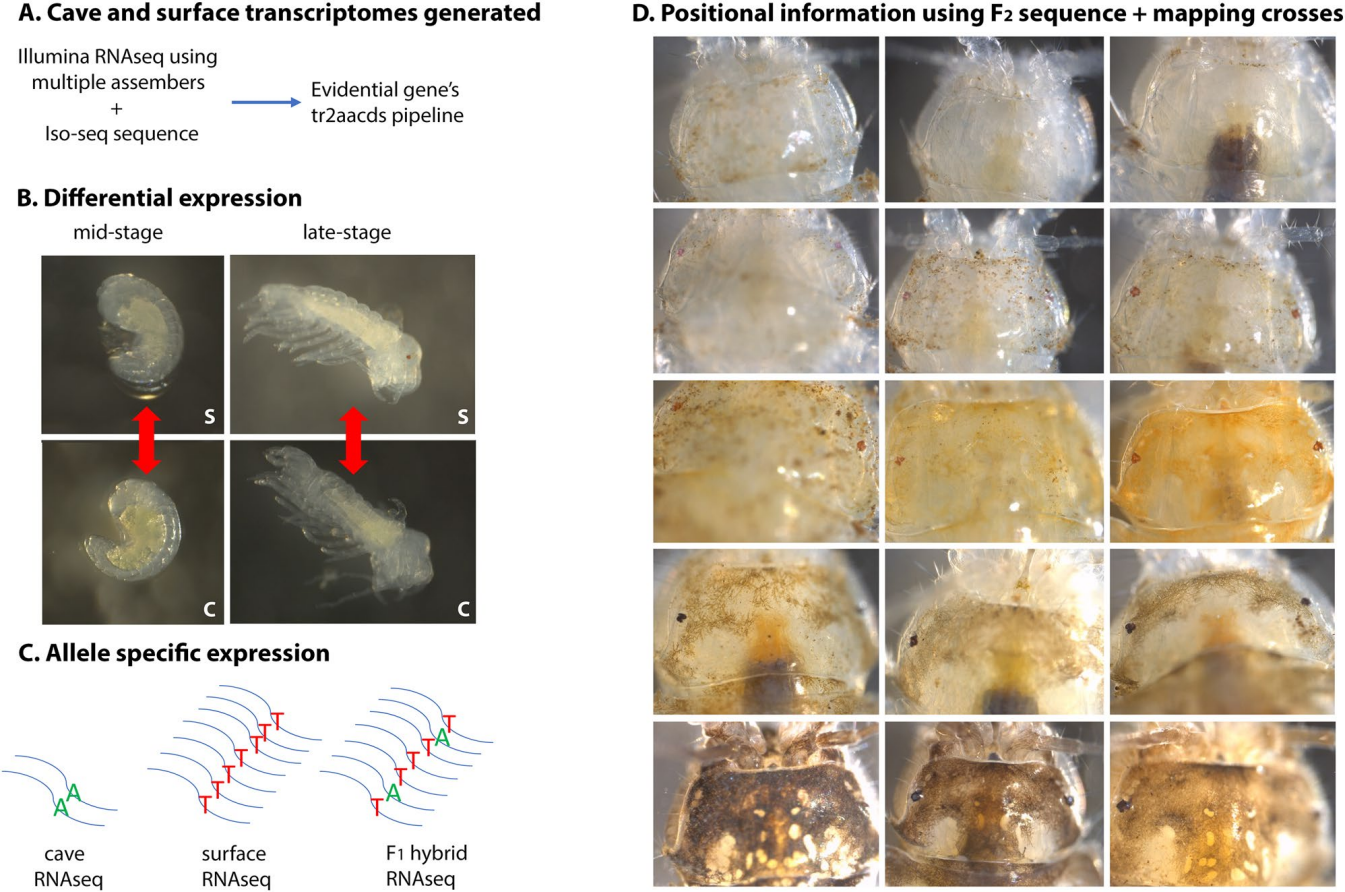
Overview of approaches. **A** Illumina RNAseq with multiple assemblers (NCGAS transcriptome pipeline) was combined with Iso-seq sequence. Then, the sequences were run through Evidential gene’s tr2aacds pipeline to generate cave and surface transcriptomes. **B** Differential expression was performed of cave, C, versus surface embryos, S, at two different stages (mid-stage and late-stage). **C** Allele-specific expression was examined to highlight genes that could have *cis*-regulatory mutations. “A” is the cave allele of a transcript and “T” is the surface allele in the example. **D** Positional information was generated using RNAseq of *F*_2_ individuals and mapping of a backcross between cave and surface population. Pictured are all 15 *F*_2_ individuals sequenced. Row 1 from left to right: MP17, MP13, and MP12. Row 2: MP10, MP11, and MP9. Row 3: MP8, MP7, and MP6. Row 4: MP4, MP5, MP3. Row 5 MP1, MP2, and MP1223 (sample phenotypes are described in Additional file 1: Table S4). A subset of these individuals were previously phenotyped [16]

Surface and cave transcriptomes of the Rakov Škocjan (SURF_rs) and Rak Channel of Planina Cave (CAVE_rr), had previously been generated using Illumina sequencing samples of a single late-stage embryonic time point [9]. The late-stage time point is when forming ommatidia can be seen and there is both eye and head pigmentation [12], Fig. 1). In order to improve these transcriptomes, Iso-seq data was obtained for one late-stage embryonic SURF_rs sample and one late-stage CAVE_rr sample [13]. Samples were also sequenced at an additional, mid-stage, embryonic time point (Fig. 1, Additional file 1: Table S1). The mid-stage time point is just before eye pigmentation is first visible in the surface form [12], Fig. 1). Several transcriptomes were made: a CAVE_rr transcriptome just from Illumina reads, a SURF_rs transcriptome just from Illumina reads, a CAVE_rr transcriptome combining Illumina reads and Iso-seq data, and a SURF_rs transcriptome combining Illumina reads and Iso-seq data. Results from the “Illumina only” or “combined” transcriptomes were similar though the combined transcriptomes contained slightly more transcripts and a slightly higher N50 (Table 1). Complete BUSCO scores were very similar with all transcriptomes scoring between 92 and 94% (Table 1). For further analyses of differential expression and allele-specific expression, we decided to move forward with the combined transcriptomes, the CAVE_rr which had 92,033 sequences and the SURF_rs which had 61,743 sequences.

**Table 1:** Quast and BUSCO output for combined (Illumina + Iso-seq) and Illumina and Iso-seq SURF_rs and CAVE_rr transcriptomes

	SURF_rs combined	SURF_rs Illumina	SURF_rs Iso-seq	CAVE_rr combined	CAVE_rr Illumina	CAVE_rr Iso-seq
# contigs (> = 0 bp)	61743	61866	37405	92033	92143	36483
# contigs (> = 1000 bp)	18027	17765	32916	18984	18490	32313
Largest contig	44632	44632	32916	39120	39120	11122
Total length (> = 0 bp)	71559147	70933662	93091806	83968993	82698998	91787813
Total length (> = 1000 bp)	54829847	54022883	90107584	56203156	54646794	88944721
N50	3265	3263	3015	3022	2990	3054
GC (%)	36.55	36.55	36.30%	37.78	37.79	36.13
# N's per 100 kbp	923.99	978	0	906.17	975.97	0
Complete BUSCOs	93.70%	93.50%	78.40%	92%	92%	79.2%
Complete and single-copy BUSCOs	90.60%	90.50%	31.20%	88.80%	88.60%	32.20%
Complete and duplicated BUSCOs	3.10%	3%	47%	3.20%	3.40%	47.00%
Fragmented BUSCOs	0.30%	0.40%	2.80%	0.40%	0.40%	3.20%
Missing BUSCOs	6.00%	6.10%	18.80%	7.60%	7.60%	17.60%

### Cave-biased and surface-biased genes identified for two embryonic timepoints

The following comparisons were performed: Late-stage CAVE_rr versus late-stage SURF_rs and mid-stage CAVE_rr versus mid-stage SURF_rs. 105 genes had higher mRNA levels in cave samples as compared to surface samples (cave-biased in expression) and 95 genes had higher mRNA levels in surface samples as compared to cave samples (surface-biased in expression) for the late-stage time point (Additional file 2: File 1). For the mid-stage time point, 55 genes were cave-biased in expression and 187 genes were surface-biased in expression (Additional file 2: File 2).

To investigate the subset of genes that might be involved in eye and pigmentation phenotypes, we searched through the differentially expressed genes for the light interaction toolkit genes, which are a set of genes that are involved in generating or maintaining the eye [14]. Within the mid-stage comparison, there were genes that were differentially expressed in melanin synthesis, pterin synthesis, heme synthesis, photo-transduction, and retinal determination network (Table 2). Within the late-stage comparison, there were genes that were differentially expressed in melanin synthesis, pterin synthesis, and photo-transduction. Differentially expressed genes of particular interest include NP_001306193.1protein scarlet, NP_001139379.1dopamine N-acetyltransferase isoform 2, NP_001164084.1arrestin 2, NP_001155991.1rhodopsin 1/6-like, and XP_008198237.1 guanine nucleotide-binding protein subunit alpha homolog.

**Table 2:** Light interacting toolkit genes that are differentially expressed using both transcriptomes

Mid-stage comparison	Bias	Light interacting gene category
NP_001139379.1dopamine N-acetyltransferase isoform 2	Surface	Melanin Synthesis
NP_001306193.1protein scarlet	Surface	Ommochrome Synthesis
XP_015838749.1 xanthine dehydrogenase isoform X1	Surface	Pterin Synthesis
XP_008193416.1 ferrochelatase, mitochondrial	Surface	Heme Synthesis
XP_008192140.1 protein ovo isoform X2*	Surface	Retinal Determination Network
XP_015834662.1 dachshund homolog 1 isoform X3*	Surface	Retinal Determination Network
NP_001164084.1arrestin 2	Surface	Phototransduction
XP_015837229.1 transient receptor potential cation channel trpm isoform X16	Surface	Phototransduction
XP_008200484.2 retinal guanylyl cyclase 2 isoform X2	Surface	Phototransduction

Late-stage comparison	Bias	Light interacting gene category
NP_001139379.1dopamine N-acetyltransferase isoform 2^+^*	Surface	Melanin Synthesis
XP_015838749.1 xanthine dehydrogenase isoform X1*	Surface	Pterin Synthesis
NP_001155991.1rhodopsin 1/6-like	Surface	Phototransduction
XP_015837026.1 transient receptor potential channel pyrexia*	Cave	Phototransduction
XP_008198237.1 guanine nucleotide-binding protein subunit alpha homolog	Cave	Phototransduction

^*^Indicates *Tribolium* IDs that were expressed and present in multiple copies in at least one transcriptome at a given time point. Categories shown are those in the light interacting toolkit [14]. Bias can be surface (surface samples showed higher mRNA levels of the gene than the cave samples) or cave (cave samples showed higher mRNA levels of the gene than the surface samples). ^**+**^Indicates two paralogs of the gene were found to have surface-biased expression

### Allele-specific expression highlights genes that are biased toward the cave allele and genes that are biased toward the surface allele in ***F***_1_ hybrids

Using ASE-Tigar [15], 89 genes showed a bias toward the surface allele and 63 genes showed a bias toward the cave allele (Additional file 2: Files 3 and 4, Additional file 1: Fig. S1). We further analyzed this list to see what subset of these genes were also differentially expressed in the late-stage CAVE_rr versus SURF_rs samples. For the genes that showed a bias toward the surface allele in the *F*_1_ hybrid samples, 27 of them showed significant differential expression (*p* adjusted value of < 0.05 and a log2Fold change of 2), all with higher expression in SURF_rs samples than CAVE_rr samples. For the genes that showed a bias toward the cave allele in *F*_1_ hybrid samples, 17 of them showed significant differential expression, all with higher expression in the CAVE_rr samples than the SURF_rs samples.

**Fig. 2:**
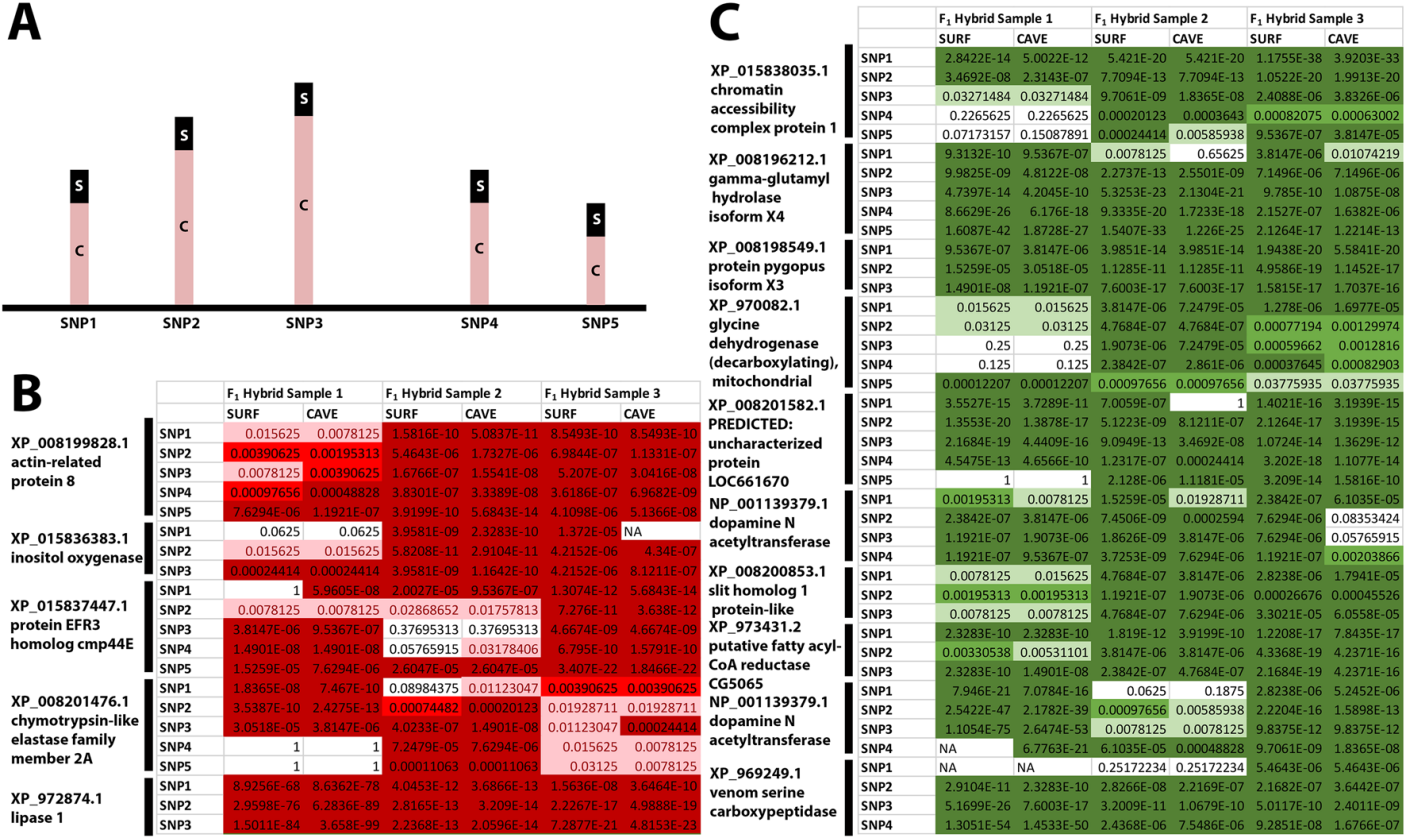
Genes with allele-specific expression. Genes that showed allele-specific expression in *F*_1_ samples using ASE-Tigar and also showed differential expression in cave versus samples (Additional file 1: Table S3; Additional file 2: Files 3 and 4) were subjected to allele counting through FreeBayes variant detector. The three *F*_1_ hybrid samples were mapped to both the surface sequence of the gene and the cave sequence of the gene. **A** The numbers of cave alleles, C, and surface alleles, S, were counted for three to five distinct SNPs along the transcript (shown as the multiple rows next to each gene name in **B**, **C**). **B** Genes biased toward the cave allele are shown in red. **C** Genes biased toward the surface allele are shown in green. A binomial distribution function was performed in Excel to detect significant deviation from the null distribution 1:1 surface to cave allele. *P-*values are shown for the binomial distribution function for each comparison of cave to surface allele. *p* > .05 is white, 0.05 > *p* > .005 is light green or light red, 0.005 > *p* > 0.0005 is green or red, *p* < 0.0005 is dark green or dark red. Genes shown below are those for which at least two SNPs per gene showed significant allele-specific expression through the binomial distribution function across all samples, regardless of whether the surface sequence or cave sequence was used as a reference. *Note* In **C**, there are two paralogues of *dopamine N-acetyltransferase*

Next, we used an allele count method for genes that showed both allele-specific expression from ASE-TIGAR and differential expression. The rationale behind this test was that one of the greatest sources of bias in allele-specific expression studies is the reference transcript used. Therefore, we wanted to count alleles using both references to make sure that an allele-specific bias was seen regardless of whether the cave or surface transcript was used. We examined the three *F*_1_ hybrid samples for five distinct SNPs, if available, along the cave and surface version of the transcript (Additional file 2: File 5; Additional file 1: Table S2). Of the 17 genes that showed bias toward the cave allele in *F*_1_ hybrids by ASE-TIGAR and differential expression in cave/surface samples, this count specific method confirmed 5 as having over-expression of the cave allele (Fig. 2; Additional file 2: File 5; Additional file 1: Table S3). Of the 35 genes that showed surface-biased allele-specific expression in *F*_1_ hybrids and differential expression in cave versus surface samples, 10 genes had allele-specific expression through allele counting. The genes that were not confirmed failed mostly because one or more of the samples showed low count numbers overall. Genes with allele-specific expression that are of particular interest are two paralogs of *dopamine N-acetyltransferase isoform 2* [blasts to *arylalkylamine N-acetyltransferase* (*aanat*)], *pygopus*, and, *efr3 homolog cmp44E*. For each gene that showed allele-specific expression, the cave and surface transcript were translated and aligned (Additional file 1: Fig. S2). Most of the genes showed a close alignment between the surface and cave version of the gene.

### Positional information for genes linked to eye and pigment regions of interest prioritizes additional candidates

To investigate the location of genes of interest, particularly those genes that showed allele-specific expression and/or differential expression (Additional file 1: Fig. S3), we performed RNA sequencing of adult *F*_2_ individuals of various phenotypes. We initially had labeled three individuals as brown, three as light brown, three as orange, three as red, and three as unpigmented. Furthermore, four of these, of different colors, were eyeless (Fig. 1). To confirm the phenotype of each adult *F*_2_ as the orange and red phenotypes are similar and the unpigmented individuals could also be red or orange as no pigment is epistatic to red and orange [8], we genotyped with the following genetic markers that mark the regions responsible for no pigment, red, and orange, respectively *disconnected*, *nckx30*, and *pax2* [8]. We found that most individuals matched with the expected genotype (Additional file 1: Table S4). However, one of the individuals initially phenotyped as red genotyped as orange. Also, another individual phenotyped as red was genotyped as both orange and red. Orange and red are two phenotypes that are similar and it is possible that additional genetic factors and/or phenotypic plasticity make the designation of orange versus red difficult at times. And finally, two of the unpigmented individuals genotyped as both unpigmented and red (Additional file 1: Table S4). For the eyeless individuals, we did not genotype the individuals as a single gene appears to be responsible and therefore the phenotyping was more straightforward [8]. We generated an updated phenotype (color by genotype), which we used moving forward (Additional file 1: Table S4). However, we note that the markers used to genotype are not the genes responsible for the respective phenotypes so recombination is possible.

ASE-Tigar was used to generate a proposed genotype for each of the 15 *F*_2_ individuals for the list of genes with cave/surface trimmed sequences (Additional file 1: Fig. S3). For each of the phenotypes, we identified all genes that had the same exact pattern of the phenotype in question. For example, for no pigment versus pigment, all genes were identified where all three unpigmented individuals were CC (two copies of the cave allele) and the 12 pigmented individuals were CS or SS (at least one copy of the cave allele). For the phenotype of no pigment versus pigment, 300 genes were identified. For the phenotype of eyeless vs eyed, 144 genes were identified. For the phenotype of orange vs not-orange, 208 were identified. To confirm if this method was accurate at identifying location of genes, we investigated whether the genes we knew to be near previously mapped regions responsible for eye and pigment loss [8] were re-identified using this method. We found that for the genes that were located in the genomic region responsible for presence versus absence of pigment, 5/8 were identified using this method (Additional file 1: Tables S5 and S6). For the genomic region responsible for orange, 2/3 were identified and finally for the eye absence phenotype, 2/3 genes were identified. For all genes that were not found, they were either missing from the transcriptome or present multiple times in the transcriptomes (therefore not able to be reliably mapped). Therefore, all genes that were present in the transcriptome and in single copy were identified, supporting that this method was accurately identifying linked regions in the genome. Regarding the phenotype of red, the method was unsuccessful probably because of recombination between the marker used, *pax2*, and the phenotype of red for the individual MP12 (when this individual, was not included, we saw 2/4 genes identified; data not shown).

To investigate the validity of the number of genes identified as linked to the three phenotypes, we permuted the data to find how many genes were linked for all possible permutations. For each phenotype, the number of linked genes was compared to the number of genes obtained for each permutation using the Wilcoxon sign-rank test with continuity correction. For all three phenotypes, *p* < 2.2e−16, indicating that the number of observed genes for each phenotype was significantly different than the number of genes obtained for the permutations. For no pigment, 300 genes were identified and all of the permutations identified between 0 and 18 genes (Additional file 1: Fig. S4; Additional file 1: List 1). For the other two phenotypes, most of the permutations yielded low numbers of the genes, but some of the permutations yielded very high numbers suggesting that these are also true locations and groups of linked genes. For example, 144 genes were identified for eye versus no-eye, and three of the other permutations of this data yielded greater than 400 genes, though the most common number of genes identified was still zero (Additional file 1: Fig. S4; Additional file 1: List 2). 208 genes were found for orange versus not-orange and four of the permutations yielded more than 100 genes (Additional file 1: Fig. S4; Additional file 1: List 3). Therefore, we conclude that this method was able to identify sets of genes that are located near one another. We do not currently have a way of determining how close they are, but future work with a chromosomal level genome will allow that question to be addressed. Here we focused on the groups of genes that are linked to mapped phenotypes of interest (no pigment, orange pigment, and eyeless), but this method can also be used to identify groups of genes that are linked elsewhere in the genome (as evidenced by the permutations that yielded large numbers of genes).

**Fig. 3:**
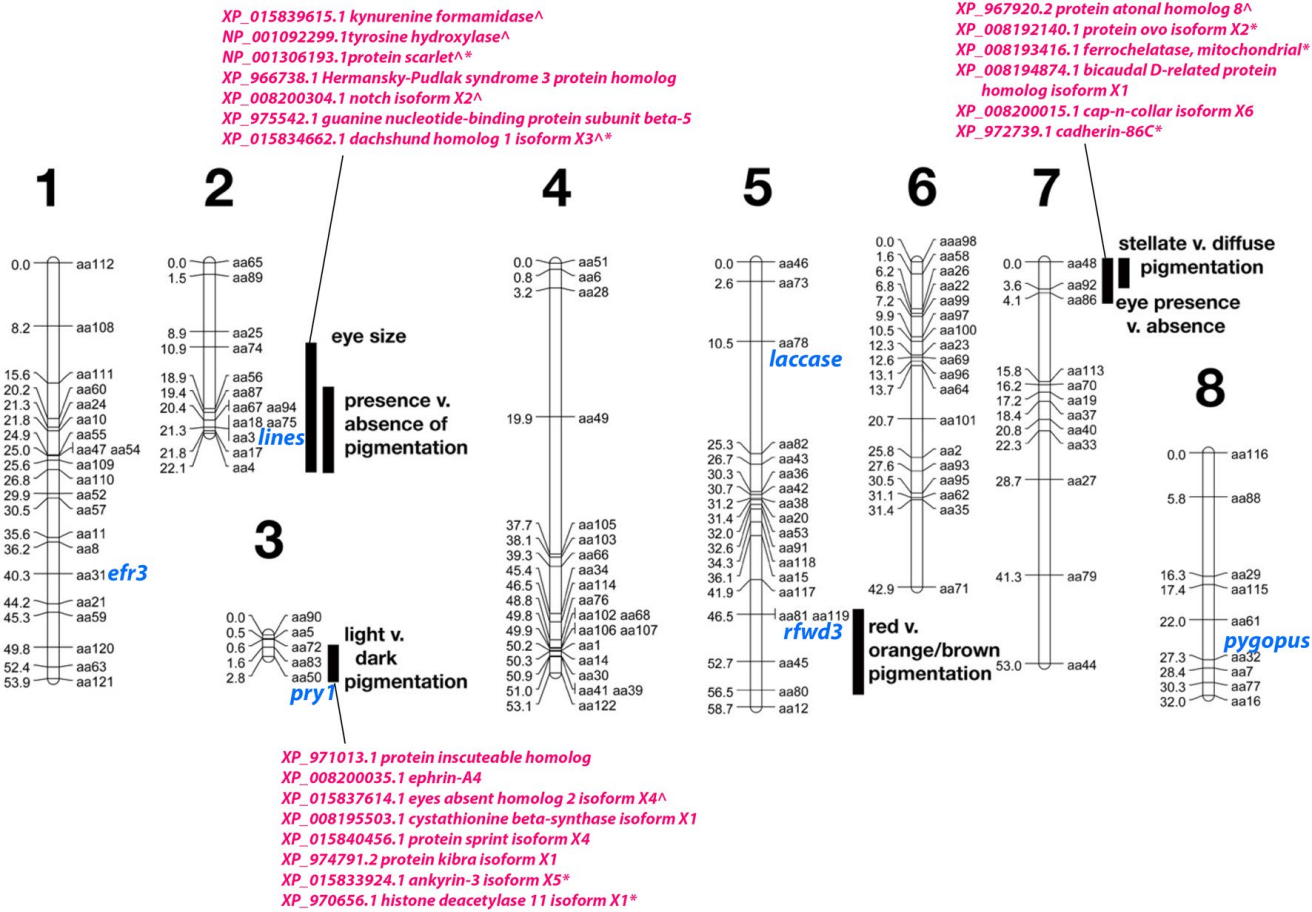
Placement of candidate genes on the genetic linkage map. 82 genes were placed near the region responsible for eye absence, 131 genes near the region responsible for orange (light/dark) and 177 genes near the region responsible for absence of pigment [8]. Shown in pink are a selection of genes from those lists. *Indicates that a gene was differentially expressed between CAVE_rr and SURF_rs samples. ^Indicates a gene that was found through our analysis that had been found also in the previous mapping analyses [8, 11]. Genes marked in blue were placed on the map by genotyping individuals from an existing backcross [8] and the gene name is present next to the genetic marker from the backcross for which there was the highest agreement

To come up with a list of genes within each region that were likely linked to the region, we selected only annotated genes and those that were present by gene ID in the list a single time. For the eyeless phenotype, there were 82 genes identified. For the region responsible for orange, there were 131 genes identified. For the region responsible for absence of pigment, there were 177 genes (Fig. 3; Additional file 2: File 6).

We selected six genes, *rfwd3*, *pry1*, *lines*, *efr3*, *laccase*, and *pygopus*, to confirm placement by genotyping an existing cross [8]. The reason we used a cross-generated from the Pivka Channel of Planina Cave (CAVE_pr) and not the Rak Channel of Planina Cave (CAVE_rr), from which our RNAseq samples were derived, was that no genome-wide mapping study has been performed yet on a cross from CAVE_rr [16]. Though CAVE_pr is thought to be distinct from CAVE_rr used to assemble the transcriptomes, the same regions were found to be responsible for eye and pigment traits in both populations. All six of these genes selected to be placed on the existing genetic map showed differential expression from either our current or previous analysis [9]. Some of these genes also showed allele-specific expression using ASE-TIGAR. Furthermore, two of the above genes were identified in the previously described positional analysis where *pry1* was proposed to be in the region linked to orange, and *lines* the region linked to presence/absence of pigment (*pry1* is not in Additional file 2: File 6 as it was present in the transcriptome in multiple copies and therefore was eliminated for that table; however, the copy of *pry1* investigated here was linked to orange in the adult *F*_2_ analysis). Genotyping of the cross showed that the location of both *pry1* and *lines* was confirmed to the region responsible for orange or presence versus absence of pigment, respectively (Additional file 1: Tables S7 and S8; Fig. 3). The other four genes were not highlighted in any of the candidate lists (*rfwd3, efr3, laccase, *and* pygopus*) and were also genotyped using the same cross and placed on the map (Additional file 1: Tables S7 and S8; Fig. 3). For genes that showed allele-specific over-expression for the cave allele, like *efr3*, the method described above through RNAseq of the *F*_2_s cannot be used as two genotypes that need to be separable, heterozygous and homozygous for the cave allele, cannot be distinguished.

## Discussion

The described transcriptomes were different from previous transcriptomes [9], in that they contained reads from an additional embryonic time point and Iso-seq sequence. Furthermore, the pipeline we used incorporated multiple assemblers. The complete BUSCO scores of the transcriptomes described here ranged from 92 to 93% which improved from our previous analysis in which the complete BUSCO scores ranged from 83 to 84% [9]. Furthermore, these updated transcriptomes had reduced representation in the fragmented and complete but duplicated categories. The combined cave transcriptome had 92,033 transcripts as compared to the surface transcriptome which had 61,743. The reduced number of transcripts could result from less successful sequencing or alignment of the cave sequences. However, these numbers are both reduced from our previous analysis with 113,000 and 119,000 respectively for surface and cave [9]. To investigate the value of including the Iso-seq data with the Illumina data, we made transcriptomes with and without the Iso-seq sequence, the inclusion of the Iso-seq sequence appears not to have greatly improved BUSCO or QUAST statistics but we imagine that the nucleotide accuracy is improved because of the Iso-seq sequence. Therefore, for all analyses, we used the combined transcriptomes.

At the mid-stage time point (just before eye pigmentation was just becoming visible and outwardly, ommatidia formation had not begun), two genes in the retinal determination network, XP_008192140.1 protein ovo isoform X2 and XP_015834662.1 dachshund homolog 1 isoform X3 showed different mRNA levels in cave and surface samples. We had expected to see more genes differentially expressed in the retinal determination network similar to studies of other arthropods [17]. It is possible that the mid-stage time point we used was either too early or too late to see major differences. Or, perhaps, the retinal determination network is mainly preserved and later events are what cause the lack of formation of ommatidia. An additional possibility is that the eye tissue is so small in *A. aquaticus* compared to the overall embryo size that it is not possible to see eye-specific results with whole embryo samples for lowly transcribed genes. Another expectation was that we would see differential expression of phototransduction genes in cave and surface samples. Aligning with our expectation, we saw lower expression of XP_015837229.1 transient receptor potential cation channel trpm isoform X16, NP_001164084.1arrestin 2, XP_008200484.2 retinal guanylyl cyclase 2 isoform X2, and NP_001155991.1rhodopsin 1/6-like. These results were similar to that in-surface and cave-dwelling spiders, which also showed differential expression of photo-transduction genes including opsins and arrestins [17]. Furthermore, decreased expression and/or accumulation of mutations of opsins has been documented in studies of many additional cave animals [18, 19].

Regarding pigmentation, we expected to see differential expression in genes involved in the ommochrome pathway as the pigments in *Asellus aquaticus* are thought to be ommochromes [20]. And in fact, *scarlet*, thought to be involved in tryptophan transport and therefore important for ommochrome pigmentation [21], showed lower mRNA levels in the cave samples as compared to the surface samples in the mid-stage time point. Interestingly, lower mRNA levels of genes in the cave samples as compared to surface samples within both melanin and pterin pathways were also seen, some at mid-stage and some at the late-stage time point. One possibility is that *A. aquaticus* could have some melanin and/or pterin pigmentation, as well as ommochrome pigmentation. Pteridines have been documented in pigmentation in other isopods [22]. Another likely possibility is that the differential expression of these genes affects other functions. For example, genes classified in the melanin pathway could be affecting production of dopamine or melatonin. Involvement of genes within the melanin pathway as players in cave-specific traits, both pigmentation and other traits, has been documented in multiple cases [23-28].

Differential expression is a way of highlighting pathways that are responsible for different phenotypes, in our case, cave-specific phenotypes. Allele-specific expression, on the other hand, can potentially identify genes that have *cis*-regulatory mutations, causative for the phenotype, rather than affected downstream pathways. Our previous allele-specific expression analysis investigated the top 50 overexpressed genes and top 50 under-expressed in cave samples as compared to surface samples [9]. Here, we investigated 14,770 genes for which we had surface/cave pairs of transcripts using an intersection of ASE-Tigar, differential expression, and an allele counting method to prioritize genes. Five genes that were cave-biased were identified including XP_015837447.1 protein EFR3 homolog cmp44E isoform X1, which had been identified in our previous analysis. *Efr3* is of interest because of its described role in hypoxia, photo-transduction, olfaction, and glucose transport [29-32]. Another cave-biased gene Blasted to lipase 1 in *Tribolium* but lipase 3 in *Homarus americanus*. Lipase 3 was recently shown to be upregulated in starved *D. melanogaster* larvae and in aged adult males suggesting a role for lipase 3 in starvation resistance and aging [33]. Ten genes showed surface-biased expression in *F*_1_ hybrids of *A. aquaticus*, including two paralogs of *dopamine N-acetyltransferase*, *aanat2* by Blast search, which has many functions including pigmentation and melatonin production [34]. Interestingly, two cavefish species were shown to have nonsense mutations in *aaad* (which is also in the melatonin synthesis pathway) and two deep sea fish had potential inactivation of *aanat2* (reviewed in [34]). Furthermore, rhythmicity of *aanat2* in *A. mexicanus* cave populations is disrupted and CRISPR mutants of *aanat2* in the surface form had reduced night-time sleep [35]. Therefore, inactivation or reduced expression of genes in the melatonin pathway might be a frequent feature in cave and deep sea animals. Another surface-biased gene in *F*_1_ hybrids was *pygopus* which in the knockout mouse had decreased insulin sensitivity and impaired lens induction [36, 37]. A previous study examined allele-specific expression in *F*_1_ hybrids of *Astyanax mexicanus* [38]. No striking overlap was seen from our list of genes with allele-specific expression and that from *A. mexicanus*.

It is likely that we missed many genes with allele-specific expression by prioritizing genes that showed allele-specific expression through multiple methods and were differentially expressed. However, we were exclusively trying to identify genes that had *cis*-regulatory mutations and did not show parent-of-origin effects. Another potential issue with the identification of genes with allele-specific expression stems from a lack of genomic information in *A. aquaticus*; we do not know whether the entire genome is diploid. One possible scenario in which part of the genome could be haploid is that *A. aquaticus* might have evolving sex chromosomes [39]. With the six genes we further genotyped to place on the map, we saw expected genotypes in the backcross, heterozygous or homozygous for the cave allele. However, one gene, NP_001155991.1rhodopsin 1/6-like showed lower expression in cave samples than surface samples and showed allele-specific expression with ASE-TIGAR but was not validated through the count method. To further investigate this gene by attempting to place it on the genetic map, we genotyped the previously published backcross from the Pivka Channel of Planina Cave [8]. Surprisingly, three genotypes were seen within the backcross, heterozygous, homozygous for cave allele, and homozygous for surface allele. A true backcross to the cave should not show individuals with a homozygous surface genotype. One possibility is that there could be haploid regions of the genome associated with sex chromosomes. Or, the cave parent could have been heterozygous for the surface allele (but this is unlikely due to the ratios of homozygous surface animals we saw). Yet another idea consistent with the presence of three genotypes is that this particular gene (or associated region) could be part of a chromosomal translocation or could be a copy number variant. Further work will need to investigate why genotyping of *rhodopsin 1/6* is inconsistent with expectations and whether there are haploid regions of the genome in *A. aquaticus*.

There are three major next steps regarding candidate genes obtained from the transcriptome analysis. The first is to obtain positional information about the candidate genes because it can inform whether a candidate shows linkage to a mapped region of interest. We were able to identify positional information for many additional genes, in particular those that are likely linked to regions responsible for pigment and eye phenotypes. When available, a chromosome level genome will provide the ultimate tool to provide positional information. Until then, the draft genome of *A. aquaticus* [7], in tandem with the methods described above, could be used to collapse scaffolds and obtain more specific positional information. The next major step is to expand the number of phenotypes that are mapped in *A. aquaticus*, ideally in multiple subterranean populations. Mapped regions are currently restricted to pigment, eye phenotypes, antennal size and body length [7, 8, 12] in a limited number of subterranean populations; there are many other phenotypes that can be examined. Finally, one other major step will be to generate a functional test, such as CRISPR, which will allow for validation of candidates of interest.

## Conclusions

Our current work expands existing transcriptomic resources in *Asellus aquaticus* to multiple embryonic time points and allows for genome-wide analysis of differential and allele-specific expression. In addition, positional information of candidates from the differential and allele-specific analysis is determined.

## Materials and methods

### Samples

For each sample, 15–89 embryos of a single brood at either 70% (mid-stage) or 90% (late-stage) of embryonic development were homogenized in 200 µl of TRIzol (Thermofisher, Waltham, MA, USA) with an Eppendorf pestle (Fig. 1). Samples were sent to the Genetic Epidemiology and Genomics Lab (GEGL), UC Berkeley, where total RNA was extracted using the RNeasy plus universal mini kit (QIAGEN). We generated sequencing libraries for three broods each of mid-stage and late-stage embryos for the Rakov Škocjan surface population (SURF_rs), mid-stage and late-stage embryos for the Rak Channel of Planina Cave population (CAVE_rr), and late-stage *F*_1_ hybrids between CAVE_rr males and SURF_rs females (HYB_rr_rs). PolyA selection was performed, and libraries were prepared using the low input protocol of the NuGEN Kit and then sequenced on the Illumina HiSeq4000 using 150 bp paired end reads at the Functional Genomics Lab, Vincent C. Coates Genomics Sequencing Laboratory, UC Berkeley. All late-stage embryonic samples used for Illumina sequencing have been previously described [9]. All samples were sequenced at a depth of 25 M reads. Two samples were prepared for Iso-seq, a single late-stage brood from SURF_rs and a single late-stage brood from CAVE_rr. These samples were extracted as described above and sequenced at the Functional Genomics Lab, Vincent C. Coates Genomics Sequencing Laboratory, UC Berkeley. Sequences are present in NCBI as (BioProject ID:PRJNA597080 and BioProject ID:PRJNA953000).

In addition to the embryonic samples outlined above, heads of 15 *F*_2_ adults generated from SURF_rs and CAVE_rr were harvested in TRIzol, extracted as described above, and sequenced on the Illumina HiSeq4000. These 15 individuals were selected based on their color and eye phenotype and included brown, light brown, red, orange, and unpigmented individuals as well as eyed and eyeless individuals (Fig. 1).

### De novo transcriptome assembly and annotation

Iso-seq output was processed through the Iso-Seq pipeline, in house, by the Functional Genomics Lab, Vincent C. Coates Genomics Sequencing Laboratory, UC Berkeley. First CCS sequences were generated, then demultiplexing/primer removal was performed, refinement was performed including polyA removal and concatemer removal, clustering was performed, and polishing. We moved forward with the polished, high-quality transcripts.

For the Illumina samples, all of the FASTQ files were first trimmed with Trimmomatic [40] using the following parameters: sliding window 4:24, headcrop 10, avgqual 30, minlen 30. Then, the NCGAS transcriptome pipeline (https://github.com/NCGAS/de-novo-transcriptome-assembly-pipeline) was used to generate separate cave and surface transcriptomes. The NCGAS transcriptome pipeline incorporates multiple assemblers: SOAP version 1.03 (kmer 35, 45, 55, 65, 75, and 85) [41], TransAbyss version 2.0.1 (kmer 35, 45, 55, 65, 75, and 85) [42], Trinity version 2.11.0 (default parameters) [43], and Velvet version 1.2.10 (kmer 35, 45, 55, 65, 75, and 85) [44]. Then, all assemblies from SOAP, TransAbyss, Trinity, and Velvet, and the Iso-seq output were combined into a single file and run through Evidential Gene’s tr2aacds pipeline [45], Fig. 1). The above steps were also performed excluding the Iso-seq output to generate Illumina only transcriptomes for comparison purposes. CAVE_rr transcriptomes and SURF_rs transcriptomes were generated. Annotation was performed using Blast2Go [46] and the *Tribolium castaneum* reference from 2019 (file entitled GCF_000002335.3_Tcas5.2_protein.faa) https://ftp.ncbi.nlm.nih.gov/genomes/all/GCF/000/002/335/GCF_000002335.3_Tcas5.2/. Make blastdb was selected, run local blast was selected, and blastx-fast was utilized.

### Quality control

BUSCO version 5.3.2 [47] and QUAST version 5.2.0 [48] were used through Galaxy [49]. For BUSCO, transcriptome assemblies (DNA) were selected and the lineage selected was Arthropoda. For QUAST, defaults were used with eukaryote selected as the type of organism.

### Differential expression

First, a Kallisto (Bioconductor version 3.12) index was made from the CAVE_rr assembly and the SURF_rs assembly [50]. Three mid-stage CAVE_rr, three late-stage CAVE_rr, three mid-stage SURF_rs, and three late-stage SURF_rs samples were mapped to both CAVE_rr and SURF_rs assemblies and quantified through Kallisto using default settings. The Kallisto output of estimated counts was combined into a single matrix of non-normalized counts. The following comparisons for differential expression were performed: late-stage CAVE_rr vs late-stage SURF_rs reads mapped to the CAVE_rr assembly, late-stage CAVE_rr vs late-stage SURF_rs reads mapped to the SURF_rs assembly, mid-stage CAVE_rr vs mid-stage SURF_rs reads mapped to the CAVE_rr assembly and mid-stage CAVE_rr vs mid-stage SURF_rs reads mapped to the SURF_rs assembly (Fig. 1). To reduce mapping inequalities due to sequence variation between the CAVE_rr and SURF_rs assemblies, we further selected genes that had different mRNA levels, here referred to as differential expression, in the same direction (e.g., lower expression in cave) using both the CAVE_rr and SURF_rs assemblies.

Only genes with reciprocal best blast hits between cave and surface transcripts were used in these comparisons to ensure homology between alleles (see section on allele-specific expression for the generation of the cave/surface pairs list). Differential expression was then performed using that matrix of counts via DEseq2 (Bioconductor version 3.12) with the default settings [51]. All differentially expressed transcripts with *p* adjusted value of < 0.05 and a log2Fold change of 2 were annotated using the Blast2GO files mentioned above. The number of times a particular gene ID was present in each output from DEseq2 was calculated. For any gene that was present in more than one copy in either the CAVE_rr or the SURF_rs transcriptome in the differentially expressed group, we had to confirm whether the multiple copies of the gene resulted from multiple paralogues or whether the transcriptome contained overlapping pieces of the same gene, present as separate transcripts. To investigate whether transcripts with the same gene ID were paralogues or the same gene, we made blast databases of the CAVE_rr and SURF_rs assemblies via European Galaxy NCBI BLAST + makeblastdb (Galaxy Version 2.10.1 + galaxy2; [52, 53]). Then, we blasted the CAVE_rr assembly to the CAVE_rr database and the SURF_rs assembly to the SURF_rs database. If a gene in the list of differentially expressed genes had a hit to a different transcript in the same transcriptome which was greater than 150 bp and > 90% identical, this gene was eliminated from the analysis as it was likely that the same gene was represented multiple times in the transcriptome. All other genes were retained except those that had gene names that were present in more than 9 copies in either the cave or the surface transcriptome and therefore likely represented multiple copies of the same gene due to high number of copies.

GO enrichment of differentially expressed genes was investigated at each time point through G:profiler [54]. The reference list of genes used for G:profiler was all genes present in nine copies or less in the output of DEseq2 for both the SURF_rs and CAVE_rr assemblies. No significant enrichment results were seen for any of the comparisons.

### Alelle-specific expression (ASE) of the ***F***_1_ hybrid samples

Allele-specific expression was performed on *F*_1_ hybrid samples (Additional file 1: Fig. S1). A blast database was created using the two transcriptomes, cave and surface, via European Galaxy NCBI BLAST + makeblastdb (Galaxy Version 2.10.1 + galaxy2; [52, 53]. Reciprocal blasting of the cave and surface transcriptomes often identified multiple hits per transcript, of which the longest and highest identity transcript was kept. Any transcript missing from blasting against either transcriptome or all those less than 400 bps was removed. The name of the surface transcript, the aligned surface sequence, the name of the cave transcript, and the aligned cave sequence were retained. Sequence files for the remaining 14,770 trimmed transcript pairs were generated for both the cave and surface transcriptomes. These sequences were combined using merge_pat_mat_fasta.pl script from ASE-TIGAR [15] resulting in a set of loci with one cave and one surface sequence (Combined transcripts).

All trimmed, pair-end reads for cave, surface, and *F*_1_ hybrid samples were mapped to the Combined transcripts using bowtie2 and ASE-TIGAR: − *X* 1000 − *k* 100—very-sensitive [55]. ASE-TIGAR generates a *Z*-value which is the number of fragments assigned to the transcript. Genes were prioritized if the *Z*-value of all cave samples mapped to the cave transcript of a gene was 3.3 × more than the *Z*-value of the cave samples mapped to the surface transcript of the gene, and the *Z*-value of all surface samples mapped to the surface transcript of a gene was 3.3 × more than the *Z*-value of the surface samples mapped to the cave transcript of the gene. Identifying these high-fidelity transcript pairs helps reduce the effect of assembly or sequencing errors and only in this subset of genes were *F*_1_ hybrid samples examined; genes showing a 3.3 × bias for one allele in all three *F*_1_ hybrid samples were kept. Finally, transcripts that weren’t able to be annotated and those that shared a *Tribolium* Id with 9 or more sequences were also removed.

### FreeBayes allele counting for the *F*_1_ embryonic hybrid samples

The goal of the following procedure was to identify SNPs which were fixed in one population and different in the other and then to investigate counts of these SNPs in all hybrid samples in genes where there were both a surface transcript and a cave transcript (Additional file 1: Fig. S1).

Reference use introduces significant bias in allele-specific expression studies, so all analyses were performed with counts against both cave and surface transcripts. Parent-of-origin effects could not be controlled for via reciprocal hybrids, as it is difficult to get cave females to breed with surface males. Instead, a subset of genes that showed allele-specific expression through ASE-TIGAR and differential expression through DEseq2 were used, as the lack of differential expression between cave and surface samples could be a signal of a parent-of-origin effect.

All late-stage SURF_rs, late-stage CAVE_rr, and *F*_1_ hybrid samples were mapped to the set of Combined transcripts (containing sets of one cave and one surface transcript) using bowtie2 [55] on Galaxy Version 2.4.2 + galaxy0 (default settings plus very fast end-to-end). Variant calling against the mapping results was performed for the cave and surface populations separately using FreeBayes [56]. All fixed SNPs (an estimated allele frequency of 100%) in cave transcripts with five or more observations were kept for cave samples, and the same for surface SNPs fixed for surface transcripts. Any SNPs that were shared between the cave and surface samples were removed, creating a final list of well-supported, fixed SNPs for both cave and surface transcripts. The three *F*_1_ hybrid samples were mapped to the Combined transcripts, and observations for each allele were retained.

For genes that showed both allele-specific expression through ASE-TIGAR and differential expression through DEseq2 (as described above), fixed SNPs were selected for both the surface and cave alleles. When the position of the SNP was not shared across the cave and surface transcripts due to indels, alignments between the alleles confirmed the diagnostic loci. Up to five SNPS that spanned the gene were selected to represent each gene of interest. The read depth for each allele at a given SNP was compiled for the *F*_1_ hybrid samples. In order to get all SNPs to be reported in the *F*_1_ hybrid samples, each *F*_1_ hybrid sample was mapped with both cave and surface samples, separately. This confirmed read depth counts, even when the sample was fixed for one allele and therefore not automatically reported as a variant.

A binomial distribution was calculated for each *F*_1_ hybrid sample using the counts against cave and surface alleles via Microsoft Office Excel (*x* = number of cave allele reads, *y* = total reads, predicted fraction = 0.5). Any gene where at least two of the five representative SNPs for that gene showed a significant (*p* < 0.05) difference between alleles in all three *F*_1_ hybrid samples was deemed to have allele-specific expression.

For any gene that was deemed to have allele-specific expression, an alignment was formed between the translated cave version of the transcript and the translated surface version of the transcript using Clustal Omega EMBL-EBI Tools [57].

### Positional information using adult *F*_2_ samples

*F*_2_ hybrid samples were used to place additional genes on the map (Additional file 1: Fig. S3). The sequences for the 15 adult *F*_2_ hybrid samples were trimmed as outlined above. Trimmed reads from the *F*_2_ hybrid samples were aligned to the Combined transcripts using ASE-TIGAR.

To determine a preliminary genotype, we divided the *Z*-value mapping to the surface transcript of a given gene by the *Z*-value mapping to the cave transcript of that same gene. As the previously mapped eye and pigment phenotypes appear to be inherited in a recessive manner [8], we were most interested in isolating homozygous cave genotypes (CC). We hypothesized that if there weren’t allele-specific expression, a homozygous cave genotype (CC) would have a *Z*-value ratio of less than 0.5 and a heterozygous (SC) or homozygous (SS) genotype (where S is the surface allele and C is the cave allele) would have a ratio of more than 0.5. For each phenotype of interest, we identified genes that had a *Z*-value ratio of more than 0.5 for SC or SS genotype and a ratio of less than 0.5 for a CC genotype. We also repeated the above procedure with two other cutoff values for a *Z*-value CC genotype (less than 0.4 and less than 0.3) and a (SC or SS) genotype (greater than 0.6 and greater than 0.7). A CC cutoff of less than 0.5 and (SC or SS) of more than 0.5 identified all nine known genes within the transcriptome, whereas the 0.4/0.6 cutoff identified only six and then 0.3/0.7 cutoff identified only three. We proceeded with the 0.5 cutoff (Additional file 1: Table S5).

We investigated three genotype patterns: (1) all orange individuals were CC, all non-orange individuals were (SC or SS); (2) all unpigmented individuals were CC and all pigmented individuals were (SC or SS); and (3) all eyeless individuals were CC and all eyed individuals were (SC or SS). To calculate the likelihood of obtaining these genotypic patterns, we first calculated the probability of each pattern where for each individual there was a ¼ chance of CC, ½ chance of SC and ½ chance of SS. The probability of obtaining the pattern of three unpigmented individuals and 12 pigmented individuals was 0.000495, the probability of obtaining the pattern of 5 orange individuals and 10 non-orange individuals was 0.000055, and the probability of obtaining the pattern of 4 eyeless individuals and 10-eyed individuals (one individual was not included as it had eye fragments and therefore was not able to be classified as eye or no-eye) was 0.0002933. To determine whether the numbers of linked genes obtained were different than what obtained when the data was permuted, all possible permutations of the data were examined. For no-pigment versus pigment, there were 455 possible permutations where three individuals were CC and twelve individuals were (SC or SS). For orange versus non-orange, 3003 permutations were possible where five individuals were CC and ten individuals were (SC or SS). For eye versus no-eye, 1001 permutations were possible where four individuals were CC and ten individuals were (SC or SS). For every possible permutation, of each phenotype, the number of genes that matched the new pattern in the permutation was identified using a script in R (Additional file 1: Script 1). A Wilcoxon ranked-sign test was performed to compare the number of matches of all of the permutations to the number of matches for the phenotype of interest.

### Genotyping

To confirm placement of candidates on the linkage map, DNA extracted from 36 individuals from an existing backcross from the Pivka Channel of Planina Cave (CAVE_pr) and the Planina Polje surface populations (SURF_pp) [8] was used to genotype and confirm the location of candidate genes. PCR was performed using 12.5 μL of GoTaq Green Master Mix (Promega), 11 μL of water, and 0.2 μL of each primer (10 μM). The genetic markers and primers that were used were for the genes *rfwd3*, *pry1*, *lines*, *efr3*, *laccase*, and *pygopus* (Additional file 1: Table S9). The PCR protocol that was used was 95 °C for 5 min, then, 35 cycles of (95 °C for 30 s, 50 °C for 30 s, 72 °C for 30 s, and finally 72 °C for 10 min). 1.5% agarose gel with SYBR Safe solution (Invitrogen) was used for visualization by gel electrophoresis. PCR products were purified using 1 μL of ExoSAP-IT (Affymetrix). The products were sent for sequencing to MCLab and visualized using Geospiza FinchTV software 1.4.0 (Geospiza, Inc.; Seattle, WA, USA; http://www.geospiza.com). Multiple sequence alignment was performed using EMBL-EBI Clustal Omega [57].

In addition, genotyping of 15 adult *F*_2_ samples (from CAVE_rr x SURF_rs) of varied phenotypes was performed for the following genes: *pax2*, *nckx30*, and *disco* using primers and methods previously described [16], Additional file 1: Table S9).

## Supplementary Information

**Additional file 1: Supplementary Table 1:** FastQC statistics for samples used for differential expression. **Supplementary Figure 1:** Allele-specific expression pipeline. **Supplementary Table 2:** Allele-specific expression in XP_008199828.1 actin-related protein 8 isoform X1 showing cave biased allele-specific expression. **Supplementary Table 3:** Genes with allele-specific expression through FreeBayes variant allele counting. **Supplementary Figure 2:** Alignments of the cave and surface proteins of genes that show allele-specific expression. **Supplementary Figure 3:** Genes linked to regions responsible for eye and pigment. **Supplementary Table 4:** Phenotype and genotype of adult F2 individuals. **Supplementary Table 5:** Ratio of cave to surface alleles used to deduce genotype of adult F2 samples. **Supplementary Table 6:** Adult F2 RNAseq confirms location of previously mapped genes near eye and pigment regions. **Supplementary Figure 4:** Permutations of the three phenotypes- pigment versus no pigment, eye versus no eye, and not-orange versus orange in the adult F2. **Supplementary List 1:** Number of genes for all possible permutations of 3 individuals CC and 12 individuals S_. **Supplementary List 2:** Number of genes for all possible permutations of 4 individuals CC and 10 individuals S_. **Supplementary List 3:** Number of genes for all possible permutations of 5 individuals CC and 10 individuals S_. **Supplementary Script 1:** Script in R to generate all permutations for no pigment/pigment where there were three no pigmented individuals and 12 pigmented individuals.

**Additional file 2: Supplementary File 1:** Late-stage differential expression, CAVE_rr vs SURF_rs reads, mapped both to CAVE_rr and SURF_rs assemblies. **Supplementary File 2:** Mid-stage differential expression, CAVE_rr vs SURF_rs reads, mapped both to CAVE_rr and SURF_rs assemblies. **Supplementary File 3:** Allele-specific expression, surface-biased, within F1 hybrids between CAVE_rr and SURF_rs populations. **Supplementary File 4:** Allele-specific expression, cave-biased, within F1 hybrids between CAVE_rr and SURF_rs populations. **Supplementary File 5:** Allele-specific expression using allele counting for three F1 hybrid samples. **Supplementary File 6:** Genes linked to regions responsible for orange pigment, no pigment, and eye loss.

## Data Availability

All sequences discussed in this report are present in the National Center for Biotechnology Information, Sequencing Reads Archive (BioProject ID:PRJNA597080 or BioProject ID:PRJNA953000).
