# Supplementary material for: Transcriptomic analysis of cave, surface, and hybrid samples of the isopod Asellus aquaticus and identification of chromosomal location of candidate genes for cave phenotype evolution
Source: EvoDevo. 2023 May 6;14:9. doi: 10.1186/s13227-023-00213-z (PMC10163715; doi:10.1186/s13227-023-00213-z)
Supplement: Supplementary file 1 — Additional file 1: Supplementary Table 1: FastQC statistics for samples used for differential expression. Supplementary Figure 1: Allele-specific expression pipeline. Supplementary Table 2: Allele-specific expression in XP_008199828.1 actin-related protein 8 isoform X1 showing cave biased allele-specific expression. Supplementary Table 3: Genes with allele-specific expression through FreeBayes variant allele counting. Supplementary Figure 2: Alignments of the cave and surface proteins of genes that show allele-specific expression. Supplementary Figure 3: Genes linked to regions responsible for eye and pigment. Supplementary Table 4: Phenotype and genotype of adult F2 individuals. Supplementary Table 5: Ratio of cave to surface alleles used to deduce genotype of adult F2 samples. Supplementary Table 6: Adult F2 RNAseq confirms location of previously mapped genes near eye and pigment regions. Supplementary Figure 4: Permutations of the three phenotypes- pigment versus no pigment, eye versus no eye, and not-orange versus orange in the adult F2. Supplementary List 1: Number of genes for all possible permutations of 3 individuals CC and 12 individuals S_. Supplementary List 2: Number of genes for all possible permutations of 4 individuals CC and 10 individuals S_. Supplementary List 3: Number of genes for all possible permutations of 5 individuals CC and 10 individuals S_. Supplementary Script 1: Script in R to generate all permutations for no pigment/pigment where there were three no pigmented individuals and 12 pigmented individuals. [file 13227_2023_213_MOESM1_ESM.docx]

**Supplementary Table 1: FastQC statistics for samples used for differential expression**

| Sample | Sample File name | Population | Stage | Number of Sequences | poor quality seqs | Seq Len | %GC |
| --- | --- | --- | --- | --- | --- | --- | --- |
| MP001 R1 | MP001_S73_L001_R1_001.fastq.gz | CAVE_rr | mid-stage | 25898783 | 0 | 151 | 31 |
| MP001 R2 | MP001_S73_L001_R2_001.fastq.gz | CAVE_rr | mid-stage | 25898783 | 0 | 151 | 29 |
| MEP6 R1 | MEP6_S74_L001_R1_001.fastq.gz | CAVE_rr | mid-stage | 27488094 | 0 | 151 | 35 |
| MEP6 R2 | MEP6_S74_L001_R2_001.fastq.gz | CAVE_rr | mid-stage | 27488094 | 0 | 151 | 32 |
| MEP7 R1 | MEP7_S75_L001_R1_001.fastq.gz | CAVE_rr | mid-stage | 29817768 | 0 | 151 | 31 |
| MEP7 R2 | MEP7_S75_L001_R2_001.fastq.gz | CAVE_rr | mid-stage | 29817768 | 0 | 151 | 30 |
| MEP8 R1 | MEP8_S76_L001_R1_001.fastq.gz | SURF_rs | mid-stage | 34235861 | 0 | 151 | 35 |
| MEP8 R2 | MEP8_S76_L001_R2_001.fastq.gz | SURF_rs | mid-stage | 34235861 | 0 | 151 | 32 |
| MEP9 R1 | MEP9_S77_L001_R1_001.fastq.gz | SURF_rs | mid-stage | 33129284 | 0 | 151 | 35 |
| MEP9 R2 | MEP9_S77_L001_R2_001.fastq.gz | SURF_rs | mid-stage | 33129284 | 0 | 151 | 32 |
| MEP10 R1 | MEP10_S78_L001_R1_001.fastq.gz | SURF_rs | mid-stage | 23072129 | 0 | 151 | 35 |
| MEP10 R2 | MEP10_S78_L001_R2_001.fastq.gz | SURF_rs | mid-stage | 23072129 | 0 | 151 | 32 |
| MPD1 R1 | MPD1_ZK34_bros_S1_R1_001.fastq.gz | CAVE_rr | late-stage | 24325226 | 0 | 150 | 35 |
| MPD1 R2 | MPD1_ZK34_bros_S1_R2_001.fastq.gz | CAVE_rr | late-stage | 24325226 | 0 | 150 | 35 |
| MPD5 R1 | MPD5_ZK_DU2_S5_R1_001.fastq.gz | CAVE_rr | late-stage | 24931978 | 0 | 150 | 34 |
| MPD5 R2 | MPD5_ZK_DU2_S5_R2_001.fastq.gz | CAVE_rr | late-stage | 24931978 | 0 | 150 | 34 |
| MPD6 R1 | MPD6_ZK_DU4_S6_R1_001.fastq.gz | CAVE_rr | late-stage | 28860312 | 0 | 150 | 35 |
| MPD6 R2 | MPD6_ZK_DU4_S6_R2_001.fastq.gz | CAVE_rr | late-stage | 28860312 | 0 | 150 | 35 |
| MPD2 R1 | MPD2_RS_DU3_S2_R1_001.fastq.gz | SURF_rs | late-stage | 24298889 | 0 | 150 | 34 |
| MPD2 R2 | MPD2_RS_DU3_S2_R2_001.fastq.gz | SURF_rs | late-stage | 24298889 | 0 | 150 | 34 |
| MPD3 R1 | MPD3_RS_DU4_S3_L006_R1_001.fastq.gz | SURF_rs | late-stage | 33164960 | 0 | 151 | 33 |
| MPD3 R2 | MPD3_RS_DU4_S3_L006_R2_001.fastq.gz | SURF_rs | late-stage | 33164960 | 0 | 151 | 34 |
| MPD8 R1 | MPD8_RSZK90_DU2_S8_L006_R1_001.fastq.gz | SURF_rs | late-stage | 32768054 | 0 | 151 | 35 |
| MPD8 R2 | MPD8_RSZK90_DU2_S8_L006_R2_001.fastq.gz | SURF_rs | late-stage | 32768054 | 0 | 151 | 36 |
| MPD4 R1 | MPD4_RSZKT3_S4_R1_001.fastq.gz | HYB_rr_rs | late-stage | 41081407 | 0 | 150 | 28 |
| MPD4 R2 | MPD4_RSZKT3_S4_R2_001.fastq.gz | HYB_rr_rs | late-stage | 41081407 | 0 | 150 | 29 |
| MPD7 R1 | MPD7_RZKT4_S7_R1_001.fastq.gz | HYB_rr_rs | late-stage | 22110561 | 0 | 150 | 33 |
| MPD7 R2 | MPD7_RZKT4_S7_R2_001.fastq.gz | HYB_rr_rs | late-stage | 22110561 | 0 | 150 | 34 |
| MPD9 R1 | MPD9_RS90_DU2_S9_R1_001.fastq.gz | HYB_rr_rs | late-stage | 24520176 | 0 | 150 | 35 |
| MPD9 R2 | MPD9_RS90_DU2_S9_R2_001.fastq.gz | HYB_rr_rs | late-stage | 24520176 | 0 | 150 | 34 |

**Supplementary Figure 1: Allele-specific expression pipeline.** A. RNAseq of 9 samples of cave, surface, and F_1_ hybrids were performed with pools of late-stage embryos. B. ASE-TIGAR (Nariai et al., 2016) was performed where first, only genes were selected where the cave samples mapped higher to the cave transcript than the surface transcript and the surface samples mapped higher to the surface transcript than the cave transcript. Also, in all three F_1_ samples, either the cave mRNA level was 3.3x higher than the surface mRNA level (cave-biased) or the surface mRNA level was 3.3x higher than the cave mRNA level (surface-biased). C. All genes from B were prioritized that also showed significant differential mRNA levels in cave versus surface samples. D. FreeBayes variant counting (Garrison and Marth, 2012) was utilized to detect the number of cave alleles and surface alleles in the F_1_ hybrid samples.

**Supplementary Table 2:** **Allele-specific expression in XP_008199828.1 actin-related protein 8 isoform X1 showing cave-biased allele-specific expression.** Three F_1_ hybrid samples were mapped to both the surface sequence of the gene and the cave sequence of the gene and the number of cave alleles and surface alleles were counted for five distinct SNPs along the transcript. In parentheses under each numbered SNP, the number before the “/” is the location of the SNP in the surface sequence and the number after the “/” is the location of the SNP in the cave sequence. (Note- there is a 19bp indel prior to the location of all of the SNPs shown). S= counts of surface allele. C=counts of cave allele. A binomial distribution function was performed to detect significant deviation from the null distribution 1:1 surface to cave allele. *P*-value is shown for each comparison of cave to surface allele.

|  | **Hybrid sample 1** | | | | | | **Hybrid sample 2** | | | | | | **Hybrid sample 3** | | | | | |
| --- | --- | --- | --- | --- | --- | --- | --- | --- | --- | --- | --- | --- | --- | --- | --- | --- | --- | --- |
|  | **mapped to surface sequence** | | | **mapped to cave sequence** | | | **mapped to surface sequence** | | | **mapped to cave sequence** | | | **mapped to surface sequence** | | | **mapped to cave sequence** | | |
|  | **S** | **C** | ***p*-value** | **C** | **S** | ***p*-value** | **S** | **C** | ***p*-value** | **C** | **S** | ***p*-value** | **S** | **C** | ***p*-value** | **C** | **S** | ***p*-value** |
| **SNP1 (855/874)** | 0 | 6 | 0.015625 | 7 | 0 | 0.0078125 | 8 | 57 | 1.5816E-10 | 59 | 8 | 5.0837E-11 | 21 | 81 | 8.5493E-10 | 81 | 21 | 8.5493E-10 |
| **SNP2 (984/1003)** | 0 | 8 | 0.00390625 | 9 | 0 | 0.00195313 | 4 | 29 | 5.4643E-06 | 31 | 4 | 1.7327E-06 | 17 | 59 | 6.9844E-07 | 63 | 17 | 1.1331E-07 |
| **SNP3 (1004/1023)** | 0 | 7 | 0.0078125 | 8 | 0 | 0.00390625 | 4 | 35 | 1.6766E-07 | 39 | 4 | 1.5541E-08 | 15 | 56 | 5.207E-07 | 62 | 15 | 3.0416E-08 |
| **SNP4 (1020/1039)** | 0 | 10 | 0.00097656 | 11 | 0 | 0.00048828 | 3 | 31 | 3.8301E-07 | 35 | 3 | 3.3389E-08 | 13 | 53 | 3.6186E-07 | 61 | 13 | 6.9682E-09 |
| **SNP5 (1065/1084)** | 0 | 17 | 7.6294E-06 | 23 | 0 | 1.1921E-07 | 2 | 39 | 3.9199E-10 | 44 | 0 | 5.6843E-14 | 12 | 46 | 4.1098E-06 | 55 | 12 | 5.1366E-08 |

**Supplementary Table 3: Genes with allele-specific expression through FreeBayes variant allele counting.** All genes shown were prioritized through ASE-Tigar and also showed differential mRNA levels in cave versus surface samples, both in reference to the surface and cave transcriptomes. In addition, FreeBayes allele counting method confirmed allele-specific expression. Allele bias is listed as surface or cave; surface indicates that that the gene has higher expression of the surface allele than the cave allele in the F_1_ hybrids and cave indicates the reciprocal. Log2FoldChange and padj are shown for the differential expression results between cave and surface late-stage samples respectively mapped to the surface and cave transcriptomes. Note- there are two different paralogues of *dopamine N acetyltransferase isoform 2*.

| gene | allele bias | SURF_rs log2FoldChange | SURF_rs padj | CAVE_rr log2FoldChange | CAVE_rr padj |
| --- | --- | --- | --- | --- | --- |
| XP_008199828.1 actin-related protein 8 isoform X1 | cave | -2.1498715 | 2.4936E-17 | -2.3960915 | 5.8427E-21 |
| XP_015836383.1 inositol oxygenase | cave | -2.4703626 | 2.2203E-07 | -2.8170622 | 1.6044E-07 |
| XP_015837447.1 protein EFR3 homolog cmp44E isoform X1 | cave | -3.4395717 | 4.5144E-25 | -3.6737863 | 2.3184E-22 |
| XP_008201476.1 chymotrypsin-like elastase family member 2A isoform X1 | cave | -2.8228706 | 7.8893E-07 | -2.7044394 | 1.769E-06 |
| XP_972874.1 lipase 1 | cave | -2.1215761 | 1.3097E-05 | -2.0365259 | 2.0625E-05 |
| XP_015838035.1 chromatin accessibility complex protein 1 | surface | 3.62527386 | 1.486E-21 | 2.4684944 | 4.5703E-10 |
| XP_008196212.1 gamma-glutamyl hydrolase isoform X4 | surface | 3.16594287 | 6.5042E-22 | 3.27368932 | 1.9883E-23 |
| XP_008198549.1 protein pygopus isoform X3 | surface | 2.92419847 | 5.5892E-31 | 2.98392565 | 3.0242E-31 |
| XP_970082.1 glycine dehydrogenase (decarboxylating), mitochondrial | surface | 2.23513327 | 1.1908E-06 | 2.41024463 | 1.0465E-07 |
| XP_008201582.1PREDICTED: uncharacterized protein LOC661670 isoform X3 | surface | 2.4780575 | 1.0996E-07 | 2.82969305 | 3.2655E-07 |
| NP_001139379.1dopamine N acetyltransferase isoform 2 | surface | 3.68607934 | 4.7174E-10 | 3.6540126 | 1.5134E-10 |
| XP_008200853.1 slit homolog 1 protein-like | surface | 4.39488558 | 5.3991E-18 | 3.79399265 | 7.881E-18 |
| XP_973431.2 putative fatty acyl-CoA reductase CG5065 | surface | 2.41026825 | 4.3557E-16 | 4.25282625 | 7.824E-32 |
| NP_001139379.1dopamine N acetyltransferase isoform 2 | surface | 4.32927609 | 7.4083E-36 | 4.36047401 | 1.341E-36 |
| XP_969249.1 venom serine carboxypeptidase | surface | 3.63307921 | 9.496E-14 | 3.73917408 | 6.3979E-15 |

**Supplementary Figure 2: Alignments of the cave and surface proteins of genes that show allele-specific expression.** Listed is gene name through Blast to the *Tribolium* genome and then in parentheses, the gene ID from the SURF_rs transcriptome and the gene ID from the CAVE_rr transcriptome. Note: these sequences are directly from the surface and cave transcriptomes and any differences were not validated through sanger sequencing.

1. XP_008199828.1 actin-related protein 8 isoform X1 (transcript_16445, TRINITY_DN244555_c1_g1_i3)

**
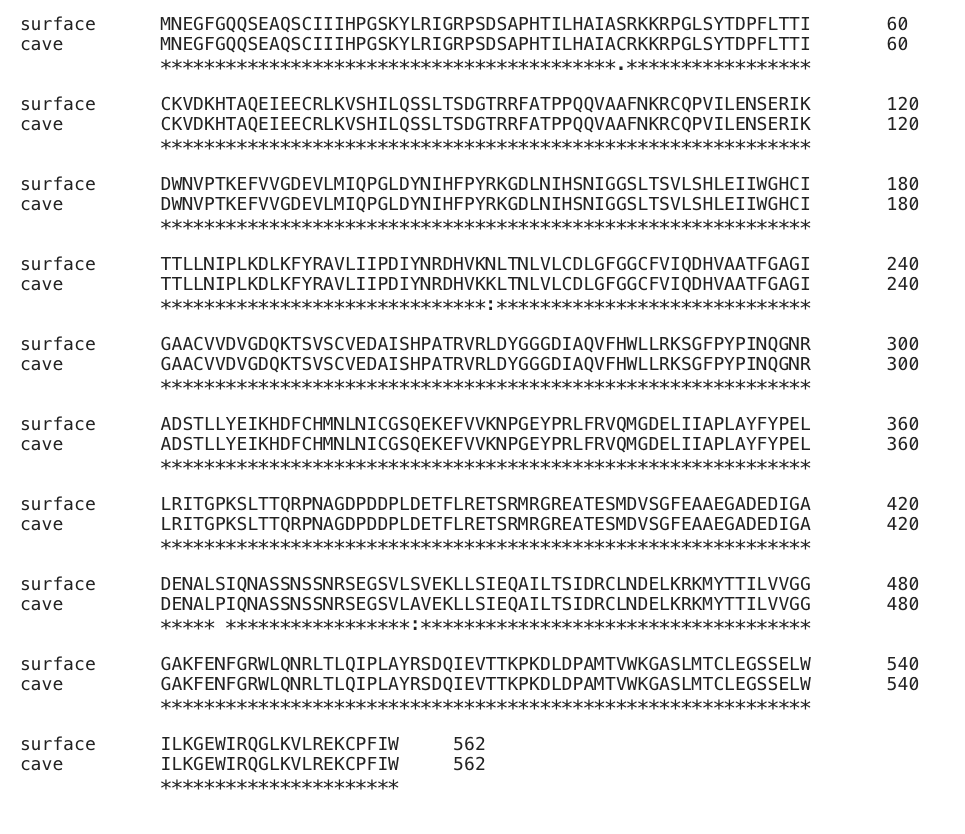
**

1. XP_015836383.1 inositol oxygenase (SOAP_k75_C655581, TransAb_k55_TransAb_k55_S3800)


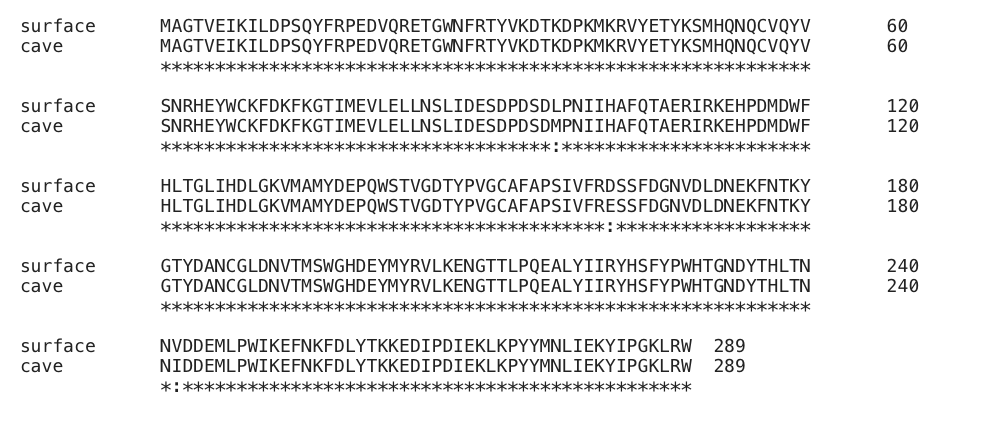


1. XP_015837447.1 protein EFR3 homolog cmp44E isoform X1 (TRINITY_DN53153_c4_g1_i10, transcript_11010)


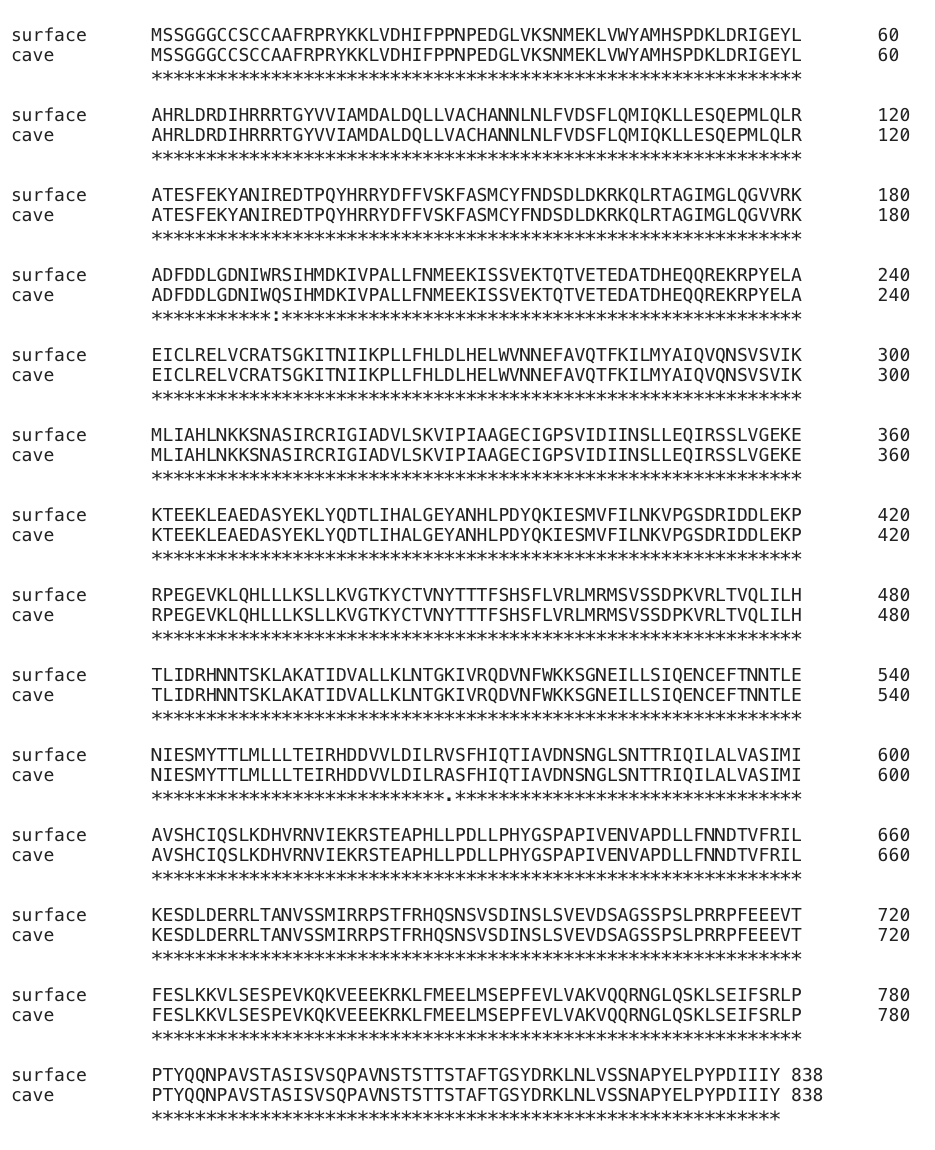


1. XP_008201476.1 chymotrypsin-like elastase family member 2A isoform X1 (Velvet_k75_Locus_34115_Transcript_11_Confidence_1.000_Length_1202, TRINITY_DN233577_c0_g1_i2)


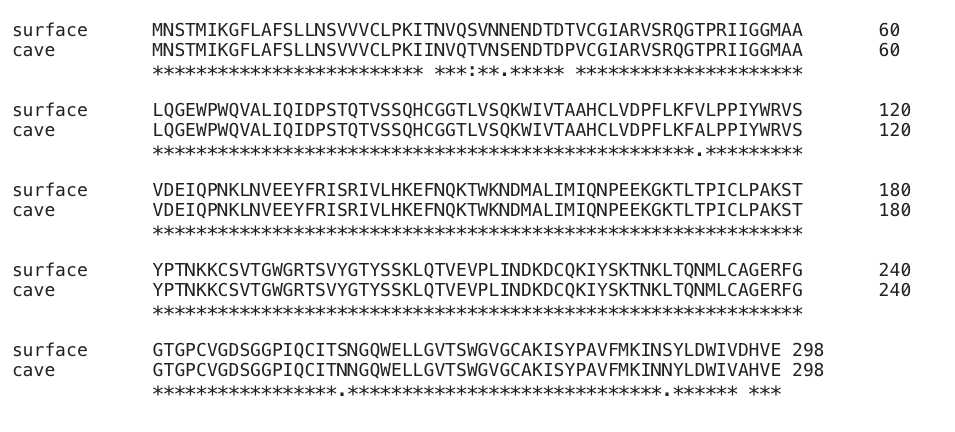


1. XP_972874.1 lipase 1 (transcript_27222, transcript_26368)


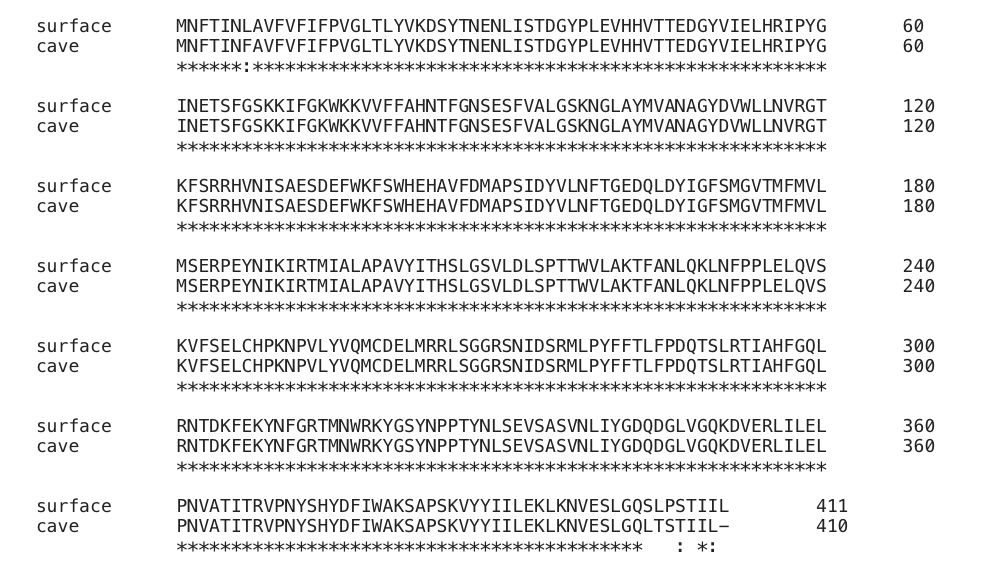


1. XP_015838035.1 chromatin accessibility complex protein 1 (TransAb_k55_S6335, TRINITY_DN247369_c7_g1_i1)


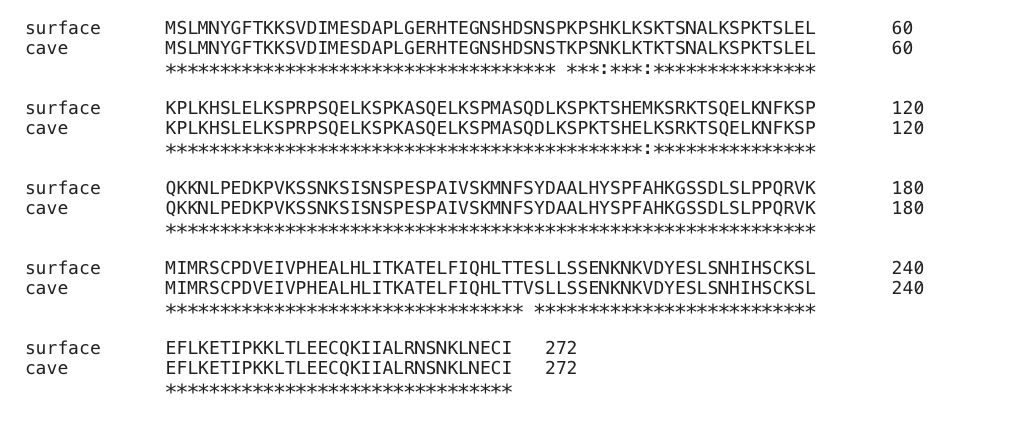


1. XP_008196212.1 gamma-glutamyl hydrolase isoform X4 (transcript_32090, SOAP_k85_C298486)


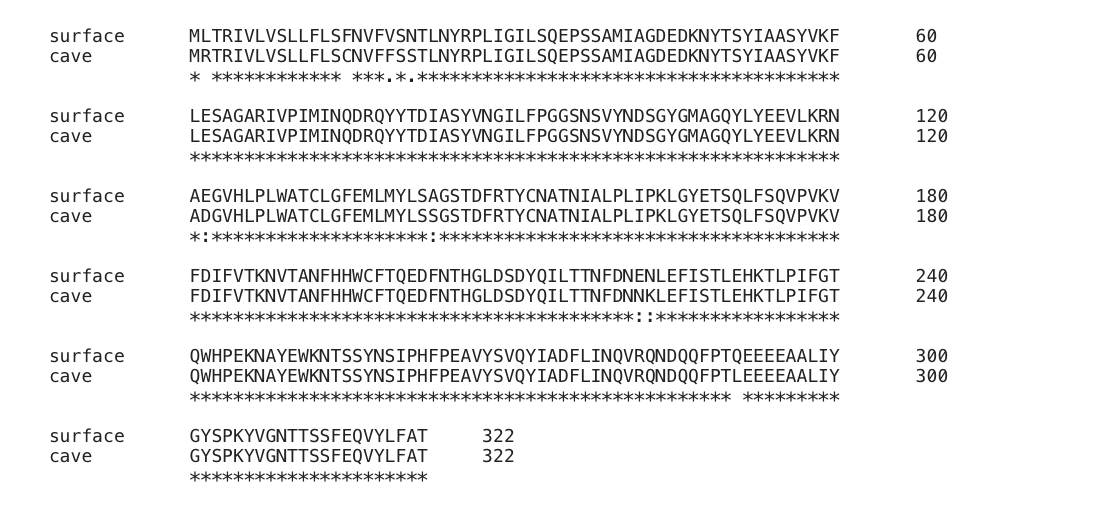


1. XP_008198549.1 protein pygopus isoform X3 (Velvet_k35_Locus_2758_Transcript_810_Confidence_0.390_Length_2527, Velvet_k75_Locus_924_Transcript_11_Confidence_1.000_Length_1830)


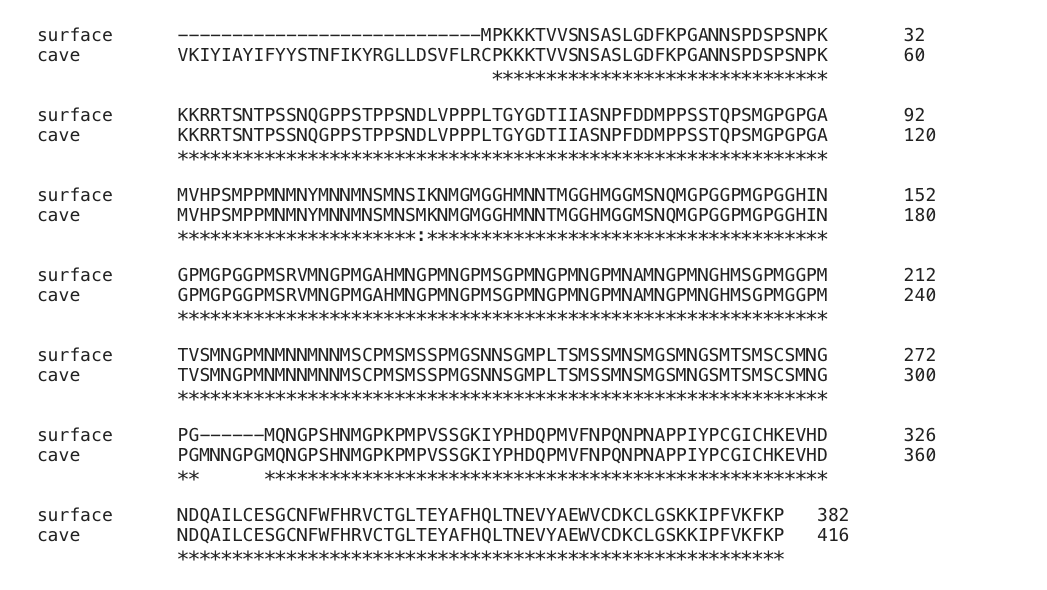


1. XP_970082.1 glycine dehydrogenase (decarboxylating), mitochondrial (transcript_9653, TRINITY_DN248464_c5_g2_i12)


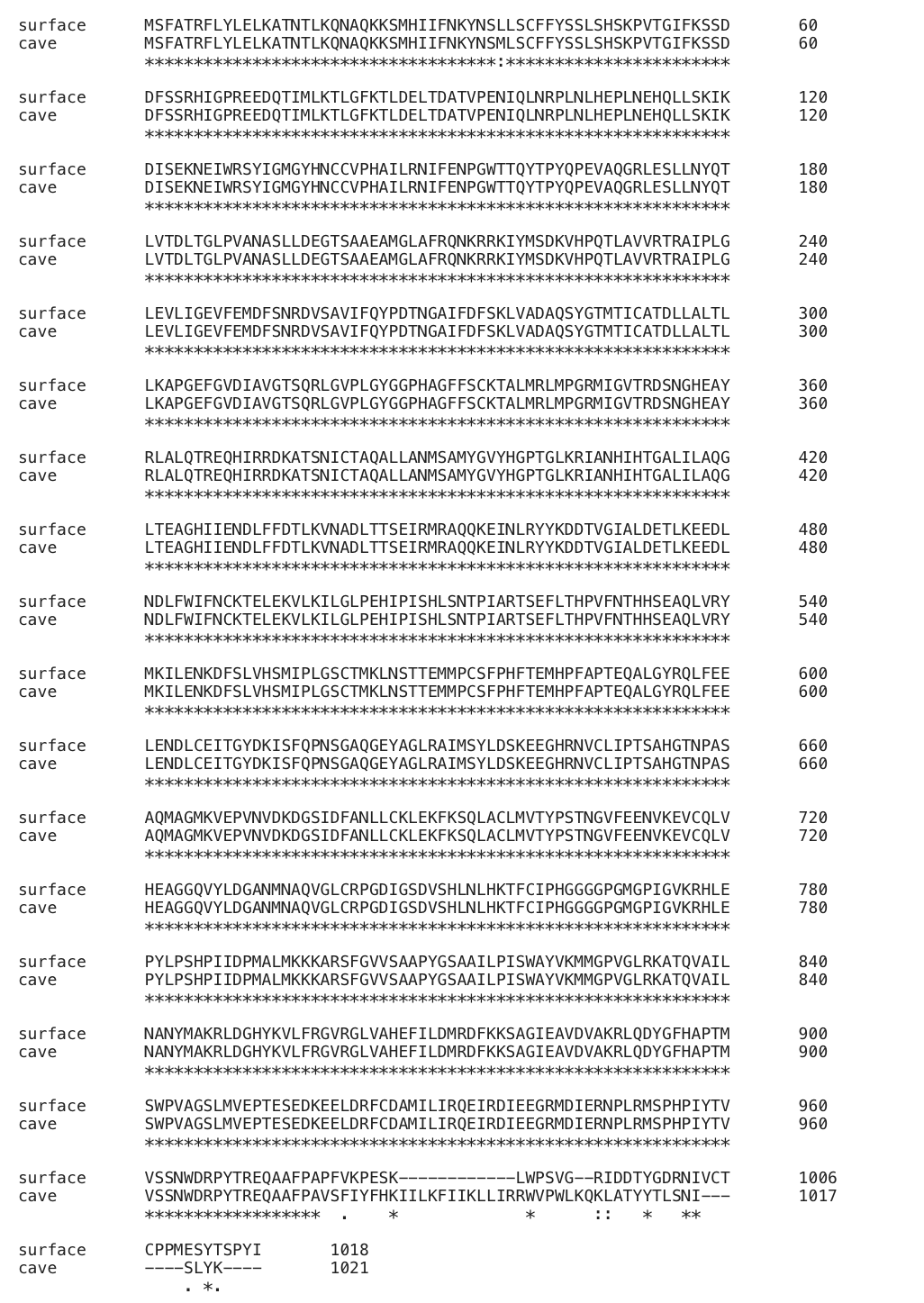


1. XP_008201582.1PREDICTED: uncharacterized protein LOC661670 isoform X3 (TRINITY_DN51132_c0_g1_i7, Velvet_k35_Locus_4997_Transcript_12_Confidence_0.500_Length_5215)


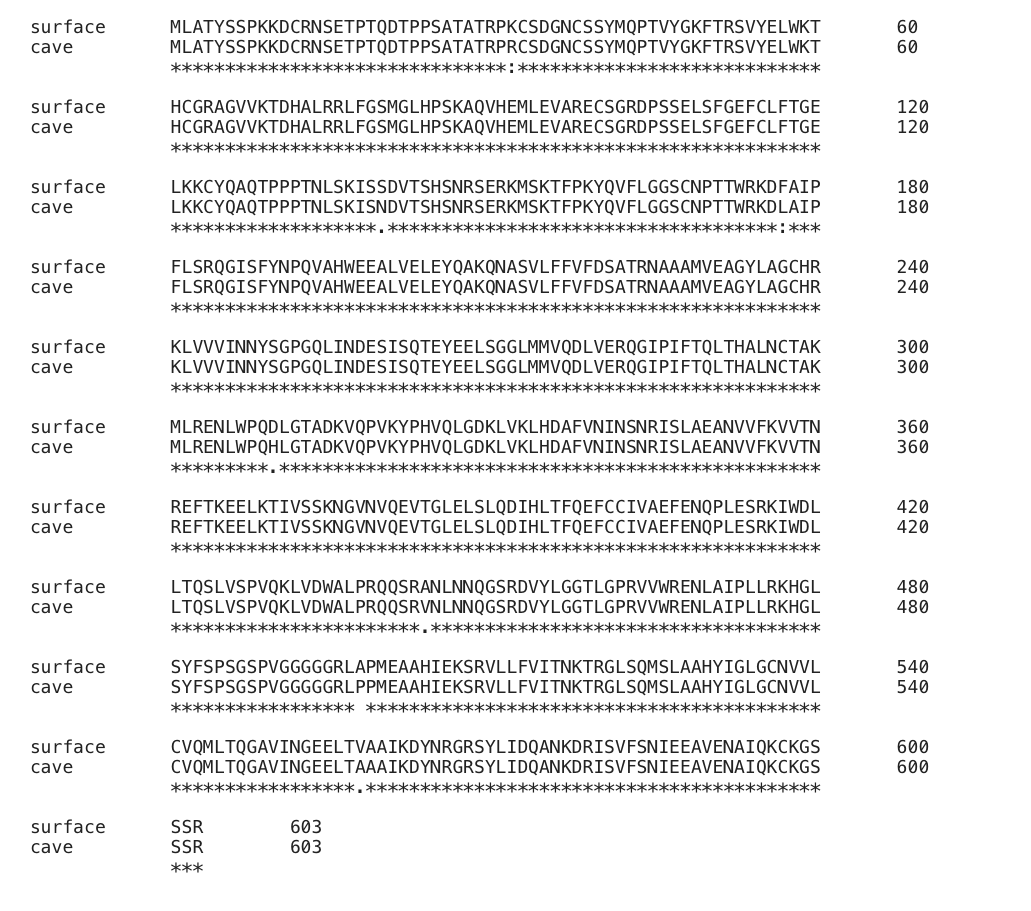


1. NP_001139379.1dopamine N acetyltransferase isoform 2 (Velvet_k65_Locus_44266_Transcript_34_Confidence_0.556_Length_2677, Velvet_k35_Locus_2766_Transcript_11_Confidence_1.000_Length_2596)


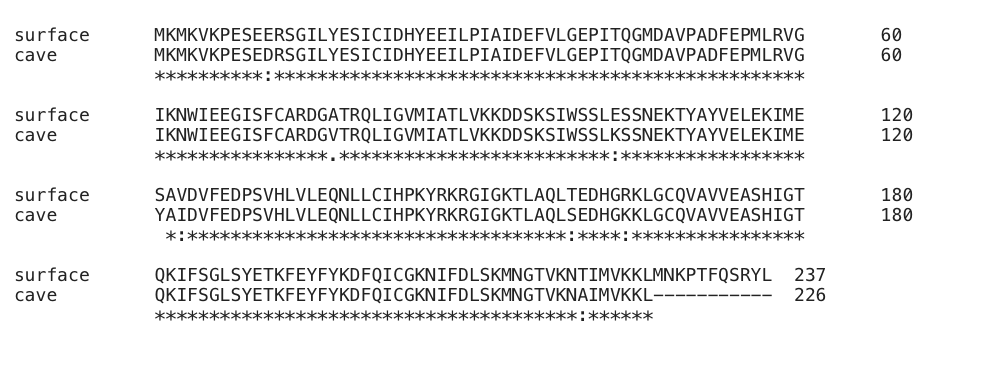


1. XP_008200853.1 slit homolog 1 protein-like (Velvet_k35_Locus_41981_Transcript_16_Confidence_0.158_Length_1683, TRINITY_DN241629_c4_g2_i1)


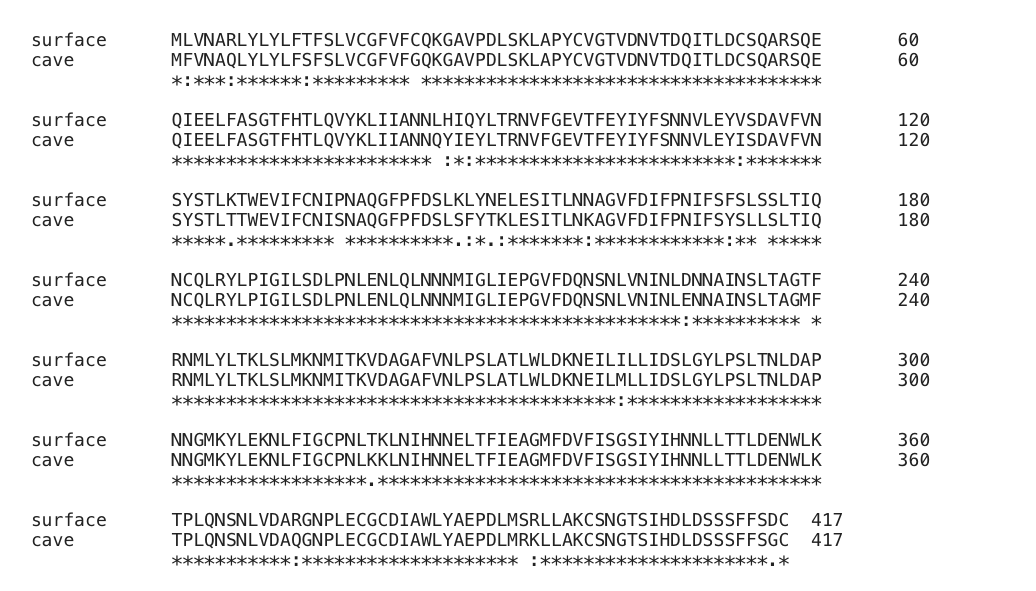


1. XP_973431.2 putative fatty acyl-CoA reductase CG5065 (SOAP_k35_C2251707, Velvet_k35_Locus_4364_Transcript_15_Confidence_0.375_Length_2203)


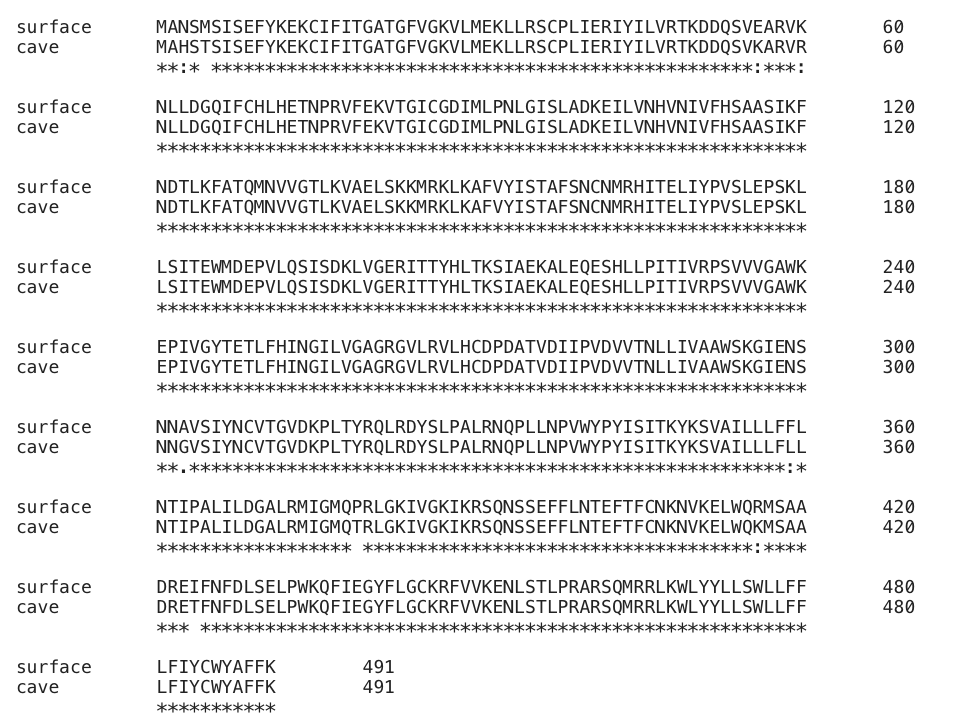


1. NP_001139379.1dopamine N acetyltransferase isoform 2 (TransAb_k45_S207109, SOAP_k35_C3647835)


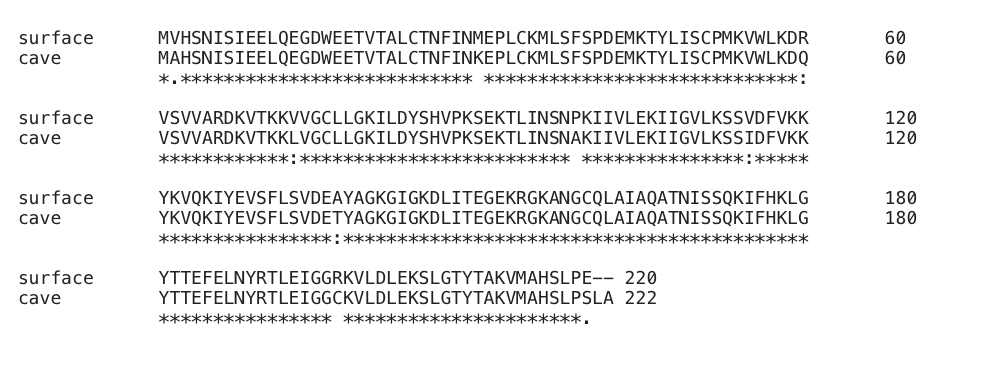


1. XP_969249.1 venom serine carboxypeptidase (TransAb_k35_S116103, SOAP_k55_C1670982)


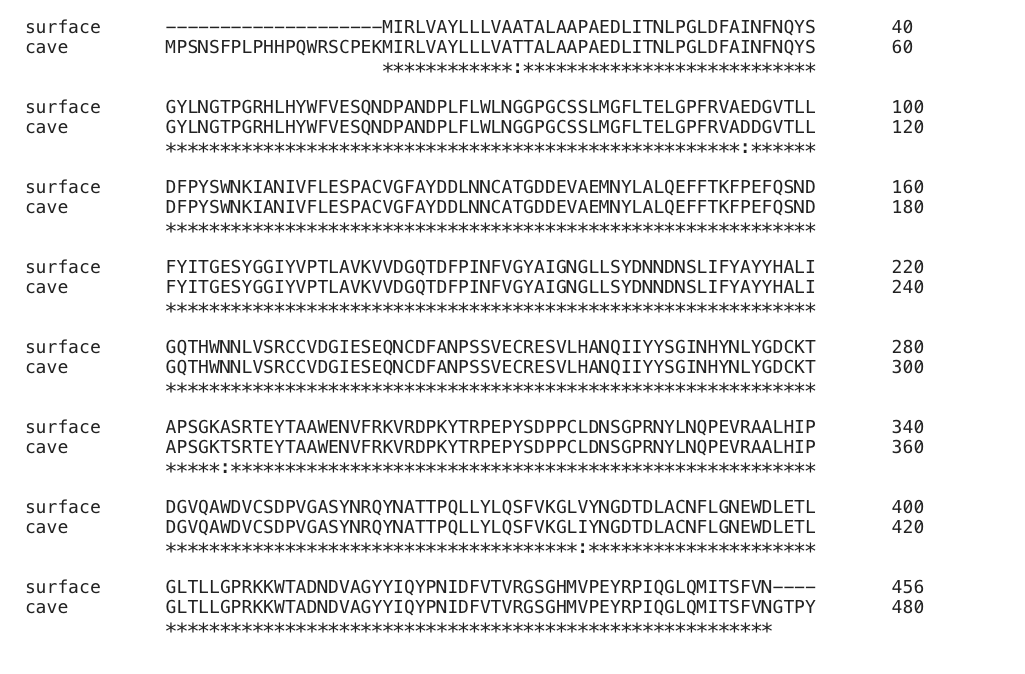


**Supplementary Figure 3: Genes linked to regions responsible for eye and pigment.** A. RNAseq of 15 adult F_2_ samples of various phenotypes were performed. B. Individuals were phenotyped and genotyped using known markers linked to different phenotypes to come up with final groupings of no pigment versus pigment, orange versus not orange, and eyed versus eyeless. C. Expression values from F_2_ animals to cave and surface transcripts using ASE-TIGAR (Nariai et al., 2016) were used to generate a genotype for each individual. When the *Z*-value of the surface allele divided by the *Z*-value of the cave allele was >0.5, the genotype was called S_ (at least one copy of the surface allele). When the *Z*-value of the surface allele divided by the *Z*-value of the cave allele was <0.5, the genotype was called CC (two copies of the cave allele).

**Supplementary Table 4: Phenotype and genotype of adult F_2_ individuals.** S=surface allele, C=cave allele. Y=yes, N=no. Photos of all individuals are present in Figure 1. ?=eye fragments but not ommatidia.

| **Sample name** | **eye color phenotype** | **eye color by genotype** | **eye** | ***disco*** | ***nckx30*** | ***pax2*** |
| --- | --- | --- | --- | --- | --- | --- |
| MP17 | no pigment | no pigment, red | ? | CC | CS | CC |
| MP12 | no pigment | no pigment | Y | CC | CS | SS |
| MP13 | no pigment | no pigment, red | N | CC | CS | CC |
| MP9 | red | orange | Y | CS | CC | CS |
| MP10 | red | orange, red | N | CS | CC | CC |
| MP11 | red | red | Y | CS | CS | CC |
| MP6 | orange | orange | Y | SS | CC | CS |
| MP7 | orange | orange | Y | CS | CC | CS |
| MP8 | orange | orange | Y | CS | CC | CS |
| MP3 | light brown | brown | Y | CS | CS | CS |
| MP4 | light brown | brown | N | CS | CS | CS |
| MP5 | light brown | brown | Y | CS | CS | CS |
| MP1223 | brown | brown | Y | CS | CS | SS |
| MP1 | brown | brown | N | CS | CS | CS |
| MP2 | brown | brown | Y | CS | CS | CS |

**Supplementary Table 5: Ratio of cave to surface alleles used to deduce genotype of adult F_2_ samples.** Three trial ratios were used. The first trial, with CC<0.5 and SC and SS>0.5, was successful at identifying all of the control genes present in the transcriptome and therefore was used for further analysis. Total genes linked to the phenotypes (in column one) are only annotated genes and ones that are found in single copy in the transcriptome.

|  | CC<.5, SC and SS>.5 | CC<.4, SC and SS>.6 | CC<.3, SC and SS>.7 |
| --- | --- | --- | --- |
| # of control genes found linked to eye loss | 2 of 2 | 1 of 2 | 1 of 2 |
| # of total genes found linked to eye loss | 82 | 58 | 48 |
| # of control genes found linked to orange | 2 of 2 | 2 of 2 | 0 of 2 |
| # of total genes found linked to orange | 131 | 98 | 66 |
| # of control genes found linked to no pigment | 5 of 5 | 3 of 5 | 2 of 5 |
| # of total genes found linked to no pigment | 177 | 141 | 113 |

**Supplementary Table 6: Adult F_2_ RNAseq confirms location of previously mapped genes near eye and pigment regions**. Y=yes linkage is confirmed through the adult F_2_ analysis, M=more than three copies of gene listed in file so can’t verify identity, NF=not found, NA=mapped name not available. Code and gene name from linkage map are from (Protas et al., 2011).

| **Phenotypic region** | **code from linkage map** | **gene name from linkage map** | **linkage** | **mapped name** |  |
| --- | --- | --- | --- | --- | --- |
| no pigment | aa87 | pale | Y | NP_001092299.1tyrosine hydroxylase | |
| no pigment | aa67 | notch | Y | XP_008200304.1 notch isoform X2 | |
| no pigment | aa18 | white1 | M | NA | |
| no pigment | aa75 | xanthine dehydrogenase | M | NA | |
| no pigment | aa3 | disconnected | NF | NA | |
| no pigment | aa17 | scarlet | Y | NP_001306193.1protein scarlet | |
| no pigment | a4 | dachshund | Y | XP_015834662.1 dachshund homolog 1 isoform X3 | |
| no pigment | aa56 | dscam | Y | XP_015836915.1 Down syndrome cell adhesion molecule-like protein Dscam2 isoform X4 | |
| orange | aa72 | dumpy | NF | NA | |
| orange | aa83 | eya | Y | XP_015837614.1 eyes absent homolog 2 isoform X4 | |
| orange | aa50 | nckx30 | Y | XP_008198217.1 sodium/potassium/calcium exchanger Nckx30C isoform X1 | |
| eyeless | aa48 | net | Y | XP_967920.2 protein atonal homolog 8 | |
| eyeless | aa92 | lim | Y | XP_008194940.1 LIM/homeobox protein Lhx5 isoform X2 | |
| eyeless | aa86 | fatfacets | NF | NA | |

**Supplementary Figure 4:** **Permutations of the three phenotypes- pigment versus no pigment, eye versus no eye, and not-orange versus orange in the adult F_2_.** A. Phenotype of pigment versus no-pigment. B. Eye versus no eye. C. Not-orange versus orange. Actual results are the number of genes linked to the known gene marking each phenotype. The number of genes/markers linked for each permutation of the data is shown with the majority of permutations showing low numbers of linked genes. For the phenotypes of no eye versus no eye and orange versus not orange there are also several additional permutations that yielded high numbers of linked genes. Wilcoxon ranked sign test comparing actual result to results of all permutations yielded p<2.2e-16 for all three phenotypes

Supplementary List 1: Number of genes for all possible permutations of 3 individuals CC and 12 individuals S_. The experimental number of genes for the phenotype of no pigment versus pigment was 300.

0, 0, 1, 6, 1, 0, 1, 3, 0, 1, 0, 0, 1, 0, 0, 1, 0, 1, 0, 0, 0, 0, 0, 0, 0, 0, 2, 1, 2, 5, 0, 0, 1, 1, 0, 0, 1, 0, 0, 0, 0, 1, 0, 0, 0, 0, 0, 0, 0, 0, 0, 3, 0, 0, 0, 0, 1, 0, 0, 0, 0, 0, 0, 2, 0, 0, 0, 1, 0, 0, 0, 1, 1, 0, 0, 1, 0, 0, 0, 1, 0, 0, 0, 0, 0, 0, 0, 0, 0, 0, 0, 0, 0, 0, 1, 0, 0, 1, 0, 0, 0, 0, 0, 1, 1, 1, 2, 0, 4, 0, 0, 0, 1, 0, 0, 0, 0, 1, 0, 0, 0, 0, 0, 0, 0, 0, 0, 0, 0, 3, 0, 0, 1, 0, 1, 0, 0, 1, 0, 0, 1, 0, 0, 0, 0, 3, 0, 0, 0, 0, 0, 0, 0, 0, 0, 0, 0, 0, 1, 1, 0, 0, 0, 0, 0, 0, 0, 0, 1, 0, 1, 0, 0, 0, 0, 0, 0, 0, 1, 0, 0, 0, 0, 0, 0, 0, 0, 0, 0, 0, 1, 0, 0, 0, 0, 0, 0, 0, 0, 0, 1, 1, 0, 1, 0, 0, 1, 0, 0, 1, 1, 0, 1, 0, 1, 1, 0, 0, 0, 1, 0, 0, 1, 1, 0, 1, 0, 0, 0, 0, 0, 2, 0, 0, 0, 0, 0, 1, 1, 0, 0, 0, 1, 0, 0, 3, 0, 0, 1, 0, 0, 2, 0, 0, 2, 0, 0, 0, 1, 0, 0, 0, 17, 1, 1, 1, 0, 0, 0, 2, 1, 0, 3, 0, 1, 0, 1, 5, 1, 0, 0, 0, 0, 2, 0, 1, 0, 0, 0, 0, 0, 0, 0, 0, 0, 0, 0, 0, 1, 0, 0, 1, 1, 0, 0, 0, 0, 2, 0, 0, 1, 0, 0, 0, 0, 0, 1, 0, 1, 1, 0, 0, 0, 1, 1, 1, 0, 0, 2, 1, 0, 0, 0, 0, 0, 1, 3, 1, 0, 1, 1, 0, 0, 0, 2, 0, 0, 3, 1, 0, 1, 0, 0, 0, 2, 0, 0, 1, 0, 0, 0, 0, 0, 0, 0, 0, 0, 0, 0, 0, 0, 0, 0, 0, 2, 1, 0, 1, 1, 0, 1, 0, 0, 0, 0, 1, 0, 1, 0, 1, 2, 0, 0, 0, 0, 0, 0, 0, 0, 1, 0, 0, 4, 0, 0, 0, 0, 0, 1, 0, 0, 0, 0, 0, 0, 0, 0, 2, 0, 0, 1, 2, 18, 1, 0, 1, 0, 0, 0, 1, 0, 1, 0, 1, 0, 0, 0, 0, 0, 0, 1, 0, 1, 1, 1, 0, 0, 0, 1, 0, 0, 0, 0, 0, 0

Supplementary List 2: Number of genes for all possible permutations of 4 individuals CC and 10 individuals S_. The experimental number of genes for the phenotype of no eye versus eye was 144.

1, 1, 1, 1, 1, 0, 1, 0, 0, 2, 3, 0, 0, 1, 0, 0, 0, 12, 0, 0, 0, 0, 0, 0, 0, 0, 0, 0, 0, 0, 0, 0, 0, 1, 0, 1, 1, 0, 0, 0, 0, 0, 0, 0, 0, 0, 0, 0, 0, 0, 0, 0, 0, 0, 0, 1, 0, 0, 0, 0, 1, 0, 0, 0, 0, 0, 456, 1, 0, 0, 1, 1, 0, 0, 2, 4, 2, 0, 1, 3, 4, 9, 5, 4, 11, 2, 0, 0, 0, 1, 1, 0, 1, 3, 0, 0, 0, 0, 2, 0, 0, 0, 0, 1, 0, 1, 0, 2, 0, 0, 0, 0, 0, 1, 1, 1, 2, 1, 3, 0, 3, 0, 0, 1, 0, 0, 0, 0, 0, 1, 0, 0, 0, 2, 1, 0, 0, 0, 0, 0, 0, 0, 0, 0, 0, 0, 0, 0, 0, 0, 0, 1, 0, 0, 0, 0, 0, 0, 1, 0, 0, 0, 2, 0, 0, 0, 0, 3, 0, 2, 0, 0, 0, 0, 0, 0, 0, 0, 0, 0, 1, 0, 0, 0, 0, 0, 1, 0, 0, 0, 0, 0, 0, 0, 0, 0, 0, 1, 1, 0, 0, 1, 0, 0, 0, 0, 0, 0, 0, 10, 0, 1, 0, 0, 0, 0, 0, 0, 2, 0, 1, 0, 0, 0, 0, 0, 0, 0, 0, 0, 0, 0, 1, 0, 0, 0, 0, 1, 0, 0, 0, 0, 0, 0, 0, 0, 0, 0, 0, 0, 0, 0, 0, 0, 0, 0, 0, 0, 0, 0, 0, 0, 0, 0, 0, 0, 0, 0, 0, 0, 1, 0, 0, 0, 0, 0, 0, 0, 0, 0, 0, 0, 0, 0, 0, 0, 0, 1, 0, 0, 0, 0, 0, 1, 0, 0, 0, 0, 0, 0, 0, 0, 0, 0, 0, 0, 0, 0, 1, 1, 0, 0, 0, 1, 0, 0, 0, 0, 0, 0, 0, 0, 0, 1, 1, 0, 0, 0, 0, 1, 0, 0, 0, 0, 0, 1, 0, 0, 1, 0, 0, 1, 0, 0, 0, 0, 0, 0, 0, 0, 0, 1, 0, 1, 0, 0, 0, 0, 1, 0, 1, 0, 0, 0, 1, 0, 0, 0, 0, 0, 0, 0, 1, 0, 0, 0, 0, 0, 0, 0, 0, 0, 0, 0, 0, 0, 0, 0, 1, 0, 1, 0, 0, 0, 0, 0, 0, 0, 0, 0, 0, 0, 0, 0, 0, 0, 0, 0, 0, 0, 0, 0, 0, 0, 0, 0, 0, 0, 0, 0, 0, 0, 1, 0, 0, 0, 0, 0, 0, 0, 0, 0, 0, 0, 0, 0, 0, 0, 0, 0, 0, 0, 1, 0, 0, 0, 0, 0, 0, 0, 0, 1, 0, 0, 0, 0, 0, 0, 1, 0, 0, 0, 0, 0, 0, 1, 0, 1, 0, 0, 0, 0, 0, 0, 0, 0, 0, 0, 0, 0, 0, 0, 0, 0, 0, 0, 0, 0, 0, 0, 0, 0, 0, 0, 0, 0, 0, 0, 0, 0, 0, 0, 0, 0, 0, 0, 0, 2, 0, 0, 0, 1, 1, 4, 0, 0, 0, 0, 0, 0, 0, 1, 0, 20, 0, 0, 0, 0, 0, 0, 2, 0, 0, 1, 0, 0, 0, 0, 1, 0, 0, 1, 0, 0, 0, 0, 0, 0, 0, 0, 0, 1, 1, 0, 0, 2, 1, 0, 0, 0, 0, 0, 0, 0, 0, 0, 0, 0, 0, 2, 1, 0, 1, 0, 0, 0, 0, 1, 0, 0, 1, 0, 0, 0, 0, 0, 0, 0, 0, 0, 0, 0, 0, 0, 1, 0, 0, 0, 0, 0, 0, 0, 0, 1, 0, 1, 0, 0, 0, 1, 1, 0, 0, 0, 0, 2, 1, 3, 570, 7, 0, 0, 0, 2, 0, 0, 1, 0, 0, 1, 0, 0, 0, 1, 0, 0, 0, 1, 411, 0, 1, 1, 0, 0, 0, 0, 1, 1, 0, 0, 0, 0, 0, 0, 0, 0, 1, 0, 1, 0, 0, 0, 0, 0, 0, 0, 0, 1, 1, 0, 0, 0, 0, 1, 0, 0, 0, 0, 1, 0, 0, 0, 0, 0, 0, 0, 0, 0, 1, 0, 0, 0, 0, 0, 0, 0, 1, 0, 0, 0, 0, 0, 0, 3, 0, 0, 0, 0, 0, 0, 0, 0, 0, 0, 0, 0, 0, 0, 0, 0, 0, 0, 0, 0, 0, 2, 5, 0, 0, 4, 0, 0, 0, 0, 0, 1, 0, 0, 0, 0, 0, 0, 0, 0, 0, 0, 0, 0, 0, 0, 0, 0, 0, 0, 0, 0, 0, 1, 1, 0, 0, 0, 0, 0, 0, 0, 1, 0, 0, 0, 0, 0, 1, 0, 0, 0, 0, 0, 0, 0, 0, 0, 0, 0, 0, 0, 0, 0, 0, 0, 0, 3, 0, 0, 0, 0, 0, 0, 55, 0, 2, 2, 1, 0, 0, 1, 0, 0, 0, 0, 0, 0, 0, 0, 0, 0, 0, 0, 0, 3, 1, 0, 3, 1, 1, 0, 0, 0, 0, 1, 0, 0, 0, 0, 0, 0, 0, 0, 0, 0, 0, 2, 2, 0, 0, 0, 0, 1, 0, 0, 0, 1, 0, 0, 0, 0, 0, 0, 0, 0, 0, 0, 0, 0, 0, 0, 0, 0, 0, 0, 0, 0, 0, 0, 0, 2, 0, 0, 0, 0, 0, 0, 0, 0, 1, 0, 0, 0, 0, 0, 0, 0, 1, 1, 0, 0, 0, 0, 0, 1, 0, 1, 1, 0, 0, 1, 0, 0, 0, 0, 0, 0, 0, 1, 0, 0, 0, 0, 1, 0, 0, 2, 0, 1, 0, 0, 0, 1, 0, 0, 0, 0, 0, 9, 3, 0, 0, 0, 0, 0, 1, 0, 0, 0, 0, 0, 0, 0, 0, 0, 3, 0, 0, 5, 0, 0, 0, 0, 0, 0, 0, 1, 0, 0, 1, 0, 0, 0, 1, 0, 1, 8, 0, 4, 2, 0, 0, 0, 0, 0, 0, 0, 0, 0, 0, 0, 0, 1, 0, 0, 1, 0, 0, 0, 0, 0, 0, 0, 0, 0, 0

Supplementary List 3: Number of genes for all possible permutations of 5 individuals CC and 10 individuals S_. The experimental number of genes for the phenotype of not-orange versus orange was 208.

1, 1, 1, 1, 1, 0, 1, 0, 0, 2, 3, 0, 0, 1, 0, 0, 0 12, 0, 0, 0, 0, 0, 0, 0, 0, 0, 0, 0, 0, 0, 0, 0, 0, 0, 1, 0, 0, 0, 0, 0, 0, 0, 0, 0, 0, 0, 0, 0, 0, 0, 0, 0, 0, 0, 0, 0, 0, 0, 0, 1, 0, 0, 0, 0, 0, 454, 1, 0, 0, 1, 1, 0, 0, 2, 4, 2, 0, 1, 1, 4, 8, 4, 3 10, 2, 0, 0, 0, 1, 1, 0, 1, 3, 0, 0, 0, 0, 2, 0, 0, 0, 0, 1, 0, 1, 0, 1, 0, 0, 0, 0, 0, 1, 1, 0, 2, 1, 3, 0, 3, 0, 0, 1, 0, 0, 0, 0, 0, 1, 0, 0, 0, 0, 1, 0, 0, 0, 0, 0, 0, 0, 0, 0, 0, 0, 0, 0, 0, 0, 0, 0, 0, 0, 0, 0, 0, 0, 1, 0, 0, 0, 1, 0, 0, 0, 0, 2, 0, 0, 0, 0, 0, 0, 0, 0, 0, 0, 0, 0, 1, 0, 0, 0, 0, 0, 0, 0, 0, 0, 0, 0, 0, 0, 0, 0, 0, 1, 142, 0, 0, 1, 0, 0, 0, 0, 0, 0, 0 10, 0, 0, 0, 0, 0, 0, 0, 0, 1, 0, 0, 0, 0, 0, 0, 0, 0, 0, 0, 0, 0, 0, 0, 0, 0, 0, 0, 0, 0, 0, 0, 0, 0, 0, 0, 0, 0, 0, 0, 0, 0, 0, 0, 0, 0, 0, 0, 0, 0, 0, 0, 0, 0, 0, 0, 0, 0, 0, 0, 0, 0, 0, 0, 0, 0, 0, 0, 0, 0, 0, 0, 0, 0, 0, 0, 0, 0, 0, 0, 0, 0, 0, 0, 0, 0, 0, 0, 0, 0, 0, 0, 0, 0, 0, 0, 0, 0, 0, 1, 1, 0, 0, 0, 1, 0, 0, 0, 0, 0, 0, 0, 0, 0, 0, 0, 0, 0, 0, 0, 0, 0, 0, 0, 0, 0, 1, 0, 0, 0, 0, 0, 0, 0, 0, 0, 0, 0, 0, 0, 0, 0, 1, 0, 1, 0, 0, 0, 0, 0, 0, 1, 0, 0, 0, 1, 0, 0, 0, 0, 0, 0, 0, 0, 0, 0, 0, 0, 0, 0, 0, 0, 0, 0, 0, 0, 0, 0, 0, 1, 0, 1, 0, 0, 0, 0, 0, 0, 0, 0, 0, 0, 0, 0, 0, 0, 0, 0, 0, 0, 0, 0, 0, 0, 0, 0, 0, 0, 0, 0, 0, 0, 0, 0, 0, 0, 0, 0, 0, 0, 0, 0, 0, 0, 0, 0, 0, 0, 0, 0, 0, 0, 0, 0, 0, 0, 0, 0, 0, 0, 0, 0, 0, 0, 0, 0, 0, 0, 0, 0, 0, 0, 0, 0, 0, 0, 0, 0, 0, 0, 0, 0, 0, 0, 0, 0, 0, 0, 0, 0, 0, 0, 0, 0, 0, 0, 0, 0, 0, 0, 0, 0, 0, 0, 0, 0, 0, 0, 0, 0, 0, 0, 0, 0, 0, 0, 0, 0, 1, 0, 0, 0, 1, 1, 3, 0, 0, 0, 0, 0, 0, 0, 0, 0, 0, 0, 0, 0, 0, 0, 0, 1, 0, 0, 1, 0, 0, 0, 0, 0, 0, 0, 1, 0, 0, 0, 0, 0, 0, 0, 0, 0, 1, 0, 0, 0, 1, 0, 0, 0, 0, 0, 0, 0, 0, 0, 0, 0, 0, 0, 1, 1, 0, 1, 0, 0, 0, 0, 0, 0, 0, 1, 0, 0, 0, 0, 0, 0, 0, 0, 0, 0, 0, 0, 0, 0, 0, 0, 0, 0, 0, 0, 0, 0, 1, 0, 1, 0, 0, 0, 0, 1, 0, 0, 0, 0, 1, 1, 2, 565, 5, 0, 0, 0, 1, 0, 0, 0, 0, 0, 1, 0, 0, 0, 0, 0, 0, 0, 1, 113, 0, 1, 0, 0, 0, 0, 0, 0, 1, 0, 0, 0, 0, 0, 0, 0, 0, 1, 0, 0, 0, 0, 0, 0, 0, 0, 0, 0, 1, 1, 0, 0, 0, 0, 0, 0, 0, 0, 0, 0, 0, 0, 0, 0, 0, 0, 0, 0, 0, 1, 0, 0, 0, 0, 0, 0, 0, 0, 0, 0, 0, 0, 0, 0, 3, 0, 0, 0, 0, 0, 0, 0, 0, 0, 0, 0, 0, 0, 0, 0, 0, 0, 0, 0, 0, 0, 0, 0, 0, 0, 0, 0, 0, 0, 0, 0, 0, 0, 0, 0, 0, 0, 0, 0, 0, 0, 0, 0, 0, 0, 0, 0, 0, 0, 0, 0, 0, 0, 0, 0, 0, 0, 0, 0, 0, 0, 0, 0, 0, 0, 0, 0, 0, 0, 0, 0, 0, 0, 0, 0, 0, 0, 0, 0, 0, 0, 0, 0, 0, 0, 0, 0, 0, 0, 0, 0, 0, 0, 0, 3, 0, 1, 0, 0, 0, 0, 0, 0, 0, 0, 0, 0, 0, 0, 0, 0, 0, 0, 0, 0, 0, 0, 0, 2, 0, 0, 0, 0, 0, 0, 1, 0, 0, 0, 0, 0, 0, 0, 0, 0, 0, 0, 1, 0, 0, 0, 0, 0, 0, 0, 0, 0, 1, 0, 0, 0, 0, 0, 0, 0, 0, 0, 0, 0, 0, 0, 0, 0, 0, 0, 0, 0, 0, 0, 0, 0, 0, 0, 0, 0, 0, 0, 0, 0, 0, 0, 0, 0, 0, 0, 0, 0, 0, 0, 0, 0, 0, 0, 0, 0, 1, 0, 0, 0, 0, 0, 0, 0, 0, 0, 0, 0, 0, 0, 0, 0, 0, 0, 0, 0, 0, 0, 0, 0, 0, 0, 0, 0, 0, 0, 0, 0, 0, 0, 0, 0, 0, 0, 0, 0, 0, 0, 0, 0, 0, 0, 0, 0, 0, 0, 0, 1, 0, 0, 0, 0, 0, 0, 0, 0, 0, 0, 0, 0, 0, 0, 0, 0, 0, 0, 0, 0, 0, 0, 0, 1, 0, 0, 0, 0, 0, 0, 0, 0, 0, 0, 0, 0, 1, 0, 0, 0, 0, 0, 0, 0, 0, 0, 0, 0, 0, 0, 0, 0, 0, 0, 0, 0, 0, 0, 0, 1, 0, 0, 0, 0, 0, 0, 0, 0, 0, 0, 0, 0, 0, 0, 0, 0, 0, 0, 0, 0, 0, 0, 0, 0, 0, 0, 0, 0, 1, 0, 0, 0, 0, 0, 0, 0, 0, 0, 0, 0, 1, 0, 0, 0, 0, 0, 0, 0, 0, 0, 0, 0, 0, 0, 0, 0, 0, 0, 0, 0, 0, 0, 0, 0, 0, 0, 0, 0, 0, 0, 0, 0, 0, 0, 0, 0, 0, 0, 0, 0, 0, 0, 0, 0, 0, 0, 0, 0, 0, 0, 0, 0, 0, 0, 0, 0, 0, 0, 0, 0, 0, 0, 0, 0, 0, 0, 1, 0, 0, 0, 0, 0, 0, 0, 0, 0, 0, 0, 1, 0, 0, 0, 0, 0, 0, 0, 0, 0, 0, 0, 0, 0, 0, 0, 0, 0, 0, 0, 0, 0, 0, 0, 0, 0, 0, 1, 0, 0, 0, 0, 0, 1, 0, 0, 0, 0, 0, 1, 0, 0, 0, 0, 0, 0, 0, 0, 0, 0, 0, 0, 0, 0, 0, 0, 0, 0, 0, 0, 1, 0, 0, 0, 0, 0, 0, 0, 0, 0, 0, 0, 0, 0, 0, 0, 0, 0, 0, 0, 0, 0, 0, 0, 0, 0, 0, 0, 0, 0, 1, 0, 0, 1, 1, 2, 1, 0 13, 1, 0, 0, 0, 0, 0, 1, 0, 0, 0, 0, 0, 0, 0, 0, 0, 0, 0, 0, 0, 0, 0, 0, 0, 0, 1, 0, 0, 0, 0, 0, 0, 0, 0, 0, 0, 0, 0, 0, 0, 1, 0, 97, 1, 0, 0, 0, 0, 0, 1, 0, 0, 0, 0, 2, 1, 0, 0, 0, 0, 0, 1, 0, 0, 0, 0, 0, 0, 0, 0, 0, 0, 0, 0, 0, 0, 0, 0, 0, 0, 0, 0, 0, 0, 1, 0, 0, 0, 0, 0, 0, 0, 0, 0, 0, 1, 2, 0, 0, 0, 0, 0, 0, 0, 0, 1, 0, 0, 0, 0, 0, 0, 0, 0, 0, 0, 0, 0, 0, 0, 0, 0, 0, 0, 0 16, 0, 0, 0, 0, 0, 0, 2, 0, 0, 0, 0, 0, 0, 0, 0, 0, 0, 0, 0, 0, 0, 0, 0, 0, 0, 0, 0, 0, 0, 0, 0, 0, 0, 0, 0, 0, 0, 0, 1, 0, 0, 0, 0, 0, 1, 0, 0, 0, 1, 0, 0, 0, 0, 0, 0, 0, 1, 0, 1, 0, 0, 0, 0, 0, 2, 0, 0, 0, 0, 0, 1, 0, 0, 0, 0, 0, 0, 0, 0, 0, 0, 0, 0, 0, 0, 0, 0, 0, 0, 0, 0, 0, 0, 0, 0, 0, 0, 0, 0, 0, 0, 0, 0, 0, 0, 0, 0, 0, 0, 0, 0, 0, 0, 0, 0, 0, 0, 0, 0, 0, 0, 0, 0, 0, 0, 0, 0, 0, 0, 0, 0, 0, 0, 0, 0, 0, 0, 0, 0, 0, 0, 0, 0, 0, 0, 0, 0, 0, 0, 0, 0, 1, 0, 0, 0, 0, 0, 0, 0, 3, 1, 0, 0, 0, 0, 0, 0, 0, 0, 0, 0, 0, 0, 0, 0, 1, 0, 0, 0, 1, 0, 0, 0, 0, 0, 0, 0, 0, 0, 0, 0, 0, 0, 0, 0, 0, 0, 0, 0, 0, 0, 0, 0, 0, 0, 0, 0, 0, 0, 0, 0, 0, 0, 0, 0, 0, 0, 0, 0, 0, 0, 0, 0, 0, 0, 0, 0, 0, 0, 0, 0, 0, 0, 0, 0, 0, 0, 0, 0, 0, 0, 0, 0, 0, 0, 0, 0, 0, 0, 0, 0, 0, 0, 0, 0, 0, 0, 0, 0, 0, 0, 0, 0, 0, 0, 0, 0, 0, 0, 0, 0, 0, 1, 0, 0, 0, 0, 0, 0, 0, 0, 0, 0, 0, 0, 0, 0, 0, 0, 0, 0, 0, 0, 0, 0, 0, 0, 0, 0, 0, 0, 0, 2, 0, 0, 0, 0, 0, 0, 0, 0, 0, 0, 0, 1, 0, 0, 0, 0, 0, 0, 0, 0, 0, 0, 0, 0, 0, 0, 0, 0, 0, 0, 0, 0, 0, 0, 0, 0, 0, 0, 0, 0, 0, 0, 0, 0, 0, 0, 0, 0, 0, 0, 0, 0, 0, 0, 0, 0, 0, 0, 0, 0, 0, 0, 0, 0, 0, 0, 0, 0, 1, 0, 0, 0, 0, 0, 0, 0, 0, 0, 0, 0, 0, 0, 0, 0, 0, 0, 0, 0, 0, 0, 0, 0, 0, 0, 0, 0, 0, 0, 0, 0, 0, 0, 0, 0, 0, 1, 0, 0, 0, 0, 0, 0, 0, 0, 0, 0, 0, 0, 0, 0, 0, 0, 0, 0, 0, 0, 0, 0, 0, 0, 0, 0, 0, 0, 0, 0, 0, 0, 0, 0, 0, 0, 0, 0, 0, 0, 0, 0, 0, 0, 0, 0, 0, 0, 0, 0, 0, 0, 0, 0, 0, 0, 0, 0, 0, 0, 0, 0, 0, 0, 0, 0, 1, 0, 0, 0, 1, 0, 0, 0, 0, 0, 0, 0, 0, 0, 0, 0, 0, 0, 0, 0, 0, 0, 0, 0, 0, 0, 0, 0, 0, 0, 0, 0, 0, 0, 0, 0, 0, 0, 1, 0, 0, 0, 0, 0, 0, 0, 0, 0, 0, 0, 0, 0, 0, 0, 0, 0, 0, 0, 0, 0, 0, 0, 0, 0, 0, 0, 0, 0, 0, 0, 0, 0, 0, 0, 0, 0, 0, 0, 0, 0, 0, 0, 0, 0, 0, 0, 0, 0, 0, 0, 0, 0, 0, 0, 0, 0, 0, 0, 0, 0, 1, 0, 0, 0, 0, 0, 0, 0, 0, 0, 0, 0, 0, 0, 0, 0, 0, 0, 0, 0, 0, 0, 0, 0, 0, 0, 0, 0, 0, 0, 0, 0, 0, 0, 0, 0, 0, 0, 0, 0, 0, 0, 0, 0, 0, 0, 0, 0, 0, 0, 0, 0, 0, 0, 0, 0, 0, 0, 0, 0, 0, 0, 0, 0, 0, 0, 0, 0, 0, 0, 0, 0, 0, 0, 0, 0, 0, 1, 0, 2, 0, 0, 0, 0, 1, 0, 0, 0, 0, 0, 0, 0, 0, 0, 0, 0, 0, 0, 0, 0, 0, 0, 0, 0, 0, 0, 0, 0, 0, 0, 0, 0, 0, 0, 0, 0, 0, 0, 0, 0, 0, 0, 0, 0, 0, 0, 0, 0, 0, 0, 1, 0, 0, 0, 0, 0, 0, 0, 0, 0, 0, 0, 0, 0, 0, 0, 0, 0, 0, 0, 0, 0, 0, 0, 0, 1, 0, 0, 0, 0, 0, 0, 0, 0, 0, 0, 0, 0, 0, 0, 0, 0, 0, 0, 0, 0, 0, 0, 0, 0, 0, 0, 0, 0, 0, 0, 0, 0, 0, 0, 0, 0, 0, 0, 0, 0, 0, 0, 0, 1, 0, 0, 0, 0, 2, 0, 0, 0, 0, 0, 0, 0, 0, 0, 0, 0, 0, 1, 0, 0, 0, 0, 0, 0, 0, 0, 0, 0, 0, 0, 0, 0, 0, 0, 0, 0, 0, 0, 0, 0, 0, 0, 0, 0, 0, 0, 0, 0, 0, 0, 0, 0, 0, 0, 0, 0, 0, 0, 0, 0, 0, 0, 0, 0, 0, 0, 0, 0, 0, 0, 0, 0, 0, 0, 0, 0, 0, 0, 0, 0, 0, 0, 0, 0, 0, 1, 0, 0, 0, 0, 0, 0, 0, 0, 1, 0, 0, 0, 0, 0, 0, 0, 0, 0, 0, 0, 0, 0, 0, 0, 0, 0, 0, 0, 0, 0, 0, 0, 0, 0, 0, 0, 0, 0, 0, 0, 0, 0, 0, 0, 0, 0, 0, 0, 0, 0, 0, 0, 0, 0, 0, 0, 0, 0, 0, 0, 0, 0, 2, 1, 3, 0, 0, 0, 0, 0, 0, 0, 0, 0, 0, 0, 0, 0, 0, 0, 0, 0, 0, 7, 0, 0, 0, 1, 0, 0, 0, 0, 0, 0, 0, 0, 0, 0, 0, 0, 0, 0, 1, 0, 0, 0, 0, 0, 0, 0, 0, 0, 0, 0, 0, 0, 2, 0, 0, 0, 0, 1, 1, 0, 0, 0, 0, 0, 0, 0, 0, 0, 0, 0, 1, 0, 0, 0, 1, 0, 0, 0, 0, 0, 0, 0, 1, 0, 0, 0, 0, 0, 0, 0, 0, 0, 0, 0, 0, 0, 0, 0, 0, 0, 0, 0, 0, 6, 0, 0, 0, 0, 0, 0, 0, 0, 0, 0, 0, 0, 0, 0, 0, 0, 0, 0, 0, 0, 1, 0, 0, 0, 0, 0, 0, 0, 0, 1, 0, 0, 0, 0, 1, 0, 0, 0, 0, 0, 1, 0, 0, 0, 0, 0, 0, 0, 0, 0, 0, 0, 0, 0, 0, 4, 0, 0 16, 0, 0, 0, 1, 0, 0, 0, 0, 0, 0, 0, 0, 0, 0, 0, 0, 0, 0, 0, 0, 0, 0, 0, 0, 0, 0, 0, 0, 0, 0, 0, 6, 0, 0, 0, 0, 0, 0, 0, 0, 0, 0, 0, 0, 0, 0, 0, 0, 0, 0, 0, 0, 0, 0, 0, 0, 0, 0, 0, 0, 0, 0, 0, 0, 0, 9, 0, 0, 6, 0, 0, 24, 6, 0, 0, 0, 0, 0, 0, 0, 0, 0, 0, 0, 0, 0, 0, 0, 0, 0, 0, 0, 0, 0, 0, 0, 0, 0, 0, 0, 0, 0, 0, 0, 0, 0, 2, 0, 0, 0, 0, 0, 0, 0, 0, 0, 0, 0, 0, 0, 0, 0, 0, 0, 0, 0, 0, 0, 0, 0, 1, 0, 0, 0, 0, 0, 1, 0, 0, 0, 0, 0, 0, 0, 0, 0, 0, 0, 0, 0, 0, 0, 0, 0, 0, 0, 0, 0, 0, 0, 0, 0, 0, 0, 0, 0, 0, 0, 0, 0, 0, 0, 0, 0, 0, 0, 0, 0, 0, 0, 0, 0, 0, 0, 0, 0, 0, 0, 0, 0, 0, 0, 0, 0, 0, 0, 0, 0, 0, 0, 0, 0, 0, 0, 0, 0, 0, 0, 0, 0, 0, 0, 0, 0, 0, 0, 0, 0, 0, 0, 0, 0, 0, 0, 0, 0, 0, 0, 0 310, 0, 0, 0, 0, 0, 0, 0, 0, 0, 0, 0, 1, 0, 0, 0, 0, 0, 0, 0, 0, 0, 0, 0, 0, 0, 0, 0, 0, 0, 0, 0, 0, 0, 0, 0, 0, 0, 0, 0, 0, 0, 0, 0, 0, 0, 0, 0, 0, 0, 1, 0, 0, 0, 1, 0, 1, 0, 0, 0, 0, 0, 0, 0, 0, 1, 0, 0, 0, 0, 0, 0, 0, 0, 0, 0, 0, 0, 1, 0, 0, 0, 0, 1, 0, 0, 0, 0, 0, 1, 1, 0, 0, 0, 0, 0, 1, 0, 0, 0, 0, 1, 5, 0, 16, 3, 0, 0, 0, 0, 0, 0, 0, 0, 0, 0, 0, 0, 0, 0, 0, 0, 0, 0, 0, 0, 0, 0, 1, 0, 0, 0, 0, 0, 0, 0, 0, 0, 0, 0, 0, 0, 0, 0, 0, 0, 0, 0, 0, 0, 0, 0, 0, 0, 0, 0, 0, 0, 0, 0, 0, 0, 0, 0, 0, 0, 0, 0, 0, 0, 0, 0, 0, 0, 0, 0, 0, 0, 1, 0, 0, 0, 0, 0, 0, 0, 0, 0, 0, 0, 0, 0, 0, 0, 0, 0, 0, 0, 0, 0, 0, 0, 0, 0, 0, 0, 0, 0, 0, 0, 1, 0, 1, 0, 0, 0, 0, 0, 0, 0, 0, 0, 0, 0, 0, 0, 0, 0, 0, 0, 0, 0, 0, 0, 0, 0, 0, 0, 0, 0, 0, 0, 0, 0, 0, 0, 0, 0, 0, 0, 0, 28, 0, 0, 0, 0, 0, 0, 0, 0, 0, 0, 0, 0, 1, 0, 0, 0, 0, 0, 0, 0, 0, 0, 0, 0, 0, 0, 0, 0, 0, 0, 0, 33, 1, 0, 1, 0, 0, 0, 0, 0, 0, 0, 1, 0, 0, 0, 0, 0, 0, 0, 0, 0, 1, 0, 0, 0, 0, 0, 0, 0, 1, 0, 0, 0, 0, 0, 0, 1, 0, 0, 0, 0, 0, 0, 0, 0, 0, 0, 0, 0

Supplementary Script 1: Script in R to generate all permutations for no pigment/pigment where there were three no pigmented individuals and 12 pigmented individuals

df <- read.csv("/Users/mprotas/Adult5.csv", header=FALSE)

NS <- ncol(df) - 1 # number of subjects

NG <- nrow(df) # number of genes

known_pattern <- c(1,1,1,1,1,1,1,1,1,1,1,0,1,0,0)

p <- (.25)^3*(.75)^12 # probability of a gene matching the known gene pattern of 3 and 12

expected_number_of_matches <- NG*p # theoretical number of matches to any 3 and 12 pattern

number_of_matches <- 0

print("Genes with same pattern as known gene:")

for (i in 1:NG) {

row <- as.numeric(df[i, c(2:16)])

if (identical(row, known_pattern) == TRUE) {

print(df[i,1])

number_of_matches <- number_of_matches + 1

}

}

match_count_known_gene <- number_of_matches

print("Number of matches to the pattern of the known gene: ")

print(match_count_known_gene)

prob_count_known_gene <- 1-pbinom(match_count_known_gene,size=NG,prob=p) # probability of the number

# of matches being equal to or greater than match_count_known_gene

print(paste("Probability of gene having this number of matches or more: ", prob_count_known_gene))

combinations <- function(size, choose) {

d <- do.call("expand.grid", rep(list(0:1), size))

d[rowSums(d) == choose,] # function for creating "unique permutations"

}

comb <- combinations(size=15, choose=12) # list of unique permutations of known pattern of 3 0's and 12 1's

N <- choose(15,3) # number of "unique permutations" of known pattern

match_count_perms <- vector( "integer" , N ) # create empty vector of match counts

for (k in 1:N) {

perm <- as.numeric(comb[k,]) # the k^th "unique permutations"

if ((identical(perm, known_pattern) == FALSE)) { # excludes the identity partition

number_of_matches <- 0

for (i in 1:NG) {

row <- as.numeric(df[i, c(2:16)])

if (identical(row, perm) == TRUE)

number_of_matches <- number_of_matches + 1

}

}

match_count_perms[k] <- number_of_matches # other than the known pattern

}

print("Number of matches for each non-identity permutation of with 3 and 12 pattern:")

print(match_count_perms)

**Supplementary Table 7: Genotypes of backcross individuals for six candidate genes.** 35 individuals from a backcross generated from Pivka Channel of Planina Cave and Planina Polje surface population (Protas et al., 2011) were genotyped for 6 candidate genes from the transcriptomic analysis. C indicates the cave allele and S indicates the surface allele. “-“ indicates unsuccessful genotyping.

| Individuals’ DNA | *rfwd3* genotype | *pry1* genotype | *lines* genotype | *efr3* genotype | *laccase* genotype | *pygopus* genotype |
| --- | --- | --- | --- | --- | --- | --- |
| BC8 074 | CC | CS | CC | CS | CC | CC |
| BC8 075 | CC | CS | CC | CS | CC | CS |
| BC8 076 | CS | CS | CS | CC | - | CS |
| BC8 077 | CS | CC | CC | CC | CS | CC |
| BC8 078 | CC | CS | CC | CC | CC | CS |
| BC8 080 | CS | CS | CS | CS | CS | CC |
| BC8 081 | CC | CS | CS | CC | CC | CS |
| BC8 082 | CC | CC | CS | CS | CC | CC |
| BC8 083 | CC | CC | CC | CS | CC | CS |
| BC9 084 | CS | CS | CS | CS | CS | CC |
| BC9 085 | CS | CC | CS | CS | CS | CS |
| BC9 086 | CC | CC | CS | CS | - | CC |
| BC9 087 | CS | CS | CS | CS | CS | CS |
| BC10 109 | CC | CS | CC | CC | CC | CS |
| BC10 119 | CC | CS | CC | CC | CC | - |
| BC10 121 | CS | CC | CC | CC | CC | CS |
| BC10 122 | CS | CS | CC | CS | CS | CC |
| BC20 123 | CS | CS | CC | CS | CS | CS |
| BC20 124 | CS | CS | CC | CS | CS | CC |
| BC20 125 | CS | CC | CS | CS | CS | CS |
| BC20 126 | CS | CS | CC | CS | CC | CC |
| BC20 128 | - | CC | CS | - | - | - |
| BC20 130 | CS | CC | CS | CC | CS | CC |
| BC30 150 | CS | CS | CS | - | - | - |
| BC12 153 | CS | CS | CS | CS | CC | CC |
| BC12 154 | CC | CS | CS | CC | CC | CS |
| BC12 155 | CS | CS | CS | CS | CC | CS |
| BC12 158 | CS | CS | CS | CC | CS | CS |
| BC12 162 | CS | CS | CS | CC | CC | CS |
| BC12 165 | CS | CS | CS | CS | CS | CS |
| BC12 167 | CS | CC | CS | CS | CS | CS |
| BC12 169 | CS | - | CS | CC | - | CC |
| BC12 170 | CS | CS | CS | CS | CS | - |
| BC23 184 | CS | CS | CS | CC | CS | CS |
| BC23 187 | CS | CC | CS | CS | - | CS |
| BC23 188 | CS | - | CS | - | CC | CS |

**Supplementary Table 8: Placement of candidate genes on genetic linkage map**

Genetic markers were found for *rfwd3*, *pry1*, *lines*, *efr3*, *laccase2*, and *pygopus* and were used to genotype backcross individuals and place the genes on the existing map (Protas et al., 2011). The closest genetic marker was found through comparison with existing genotyping information (Protas et al., 2011). The first number in individuals genotyped is the number of individuals that agree between the gene and closest genetic marker and the second number is the total number of individuals in which genotypes of the gene and closest genetic marker were compared. Some of these genes mapped to previously known regions responsible for red, orange, and presence/absence of pigment (Protas et al., 2011). For graphical representation, see Figure 3.

| **Gene Name** | **individuals genotyped** | **Closest genetic marker** | **Region** |
| --- | --- | --- | --- |
| *rfwd3* | 29/34 | aa81 | red |
| *pry1* | 28/29 | aa50 | orange |
| *lines* | 34/34 | aa3 | no pigment |
| *efr3* | 31/32 | aa31 | none |
| *laccase* | 29/30 | aa78 | none |
| *pygopus* | 28/31 | aa7 | none |

**Supplementary Table 9: Genotyped differences used for placement of genes on the map.** Forward and reverse primers are listed plus the genotyped difference. In brackets is the difference with the cave allele listed before the “/” and the surface allele listed after the “/”. “-“ indicates absence of a nucleotide. Markers in *pax2*, *nckx30*, and *disco* are previously described (Re et al., 2018).

|  | **Forward primer (5’-3’)** | **Reverse primer (5’-3’)** | **Genotyped Difference** |
| --- | --- | --- | --- |
| *pax2* | TACAAGAGAGAAAACCCGACGATGT | GGATAGGAGGAAGGAGTGCTGGTTT | CACTTTTACTACGTTNATAGCACT[--------------------/TTTACTACGTTGATAGCACT]GTTTTTTTGTGGATA |
| *nckx30* | TCCTCCGAGATCTGCAACTTCTCTA | GTCTTCGCTTGTCCAAATGACGATA | TCCGTGAA[A/T]ATGGAGGG |
| *disco* | AACCGCCATTCTGCTAATCC | CGCTATTCATGCTGTCTTCCA | GGCAACCC[T/A]GTTAGAGG |
| *rfwd3* | TCTGAAACGGGAATTGGAGTCACA | GCCAAATCCAGCAAACATAGCAACT | AAAAACT[A/G]AAAAA |
| *pry1* | GGGCTGATACCATGGCTGCTACC | TTGCTACGCCTACTCCCAAACGG | ACTATGA[T/C]TTCGCAA |
| *lines* | TGTCCAAGATTGATGCAACA | AGGCTGGGTGACTTTCATAT | TTTATTCCCCTATAATAT[A/T]AATGCCTGTT |
| *efr3* | TATTGTTTGCTCCCTTATTTGCA | GCTGAGTATGCCCATCTACTATG | TAGATG[C/T]GTTTAA |
| *laccase* | TGTTCTACTCTCAAGCTGGAATG | TGTGTATGTGTGTGTGCATGTAT | CCAATAAATAGTCACATA[ACATACAAAGATGTAAA/TAATATACAAAAA] |
| *pygopus* | ACAGGCTTGACGGAATATGC | TGGCATTTTCAACGACAGCT | GGGTCTGTGATAAATGTCTCGG[G/A]TCTAAAAAAATTCCGTTT |

**References:**

Protas, M.E., Trontelj, P., and Patel, N.H. 2011. Genetic basis of eye and pigment loss in the cave crustacean, Asellus aquaticus. PNAS. 108: 5702–5707.

Nariai, N., Kojima, K., Mimori, T., Kawai, Y., and Nagasaki, M. 2016. A Bayesian approach for estimating allele-specific expression from RNA-Seq data with diploid genomes. BMC Genomics. 1:7–17.

Re, C., Fišer, Ž., Perez, J., Tacdol, A., Trontelj, P., and Protas, M.E. 2018. Common genetic basis of eye and pigment loss in two distinct cave populations of the isopod crustacean Asellus aquaticus. Integr Comp Biol. 58: 421–430.

stylefix
